# Unlocking the molecular basis of wheat straw composition and morphological traits through multi-locus GWAS

**DOI:** 10.1186/s12870-022-03900-6

**Published:** 2022-11-08

**Authors:** Salvatore Esposito, Francesca Taranto, Paolo Vitale, Donatella Bianca Maria Ficco, Salvatore Antonio Colecchia, Piergiorgio Stevanato, Pasquale De Vita

**Affiliations:** 1 Research Centre for Cereal and Industrial Crops (CREA-CI), CREA - Council for Agricultural Research and Economics, 71122 Foggia, Italy; 2 Institute of Biosciences and Bioresources, (CNR-IBBR), 70126 Bari, Italy; 3 Department of the Sciences of Agriculture, Food and Environment, University of Foggia, 71122 Foggia, Italy; 4 Department of Agronomy, Food, Natural Resources, Animals and Environment, University of Padova, 35020 Padova, Legnaro Italy

**Keywords:** Acid detergent fiber, Acid detergent lignin, Biofuel, ML-GWAS, Neutral detergent fiber, QTNs

## Abstract

**Background:**

Rapid reductions in emissions from fossil fuel burning are needed to curb global climate change. Biofuel production from crop residues can contribute to reducing the energy crisis and environmental deterioration. Wheat is a renewable source for biofuels owing to the low cost and high availability of its residues. Thus, identifying candidate genes controlling these traits is pivotal for efficient biofuel production. Here, six multi-locus genome-wide association (ML-GWAS) models were applied using 185 tetraploid wheat accessions to detect quantitative trait nucleotides (QTNs) for fifteen traits associated with biomass composition.

**Results:**

Among the 470 QTNs, only 72 identified by at least two models were considered as reliable. Among these latter, 16 also showed a significant effect on the corresponding trait (*p*.value < 0.05). Candidate genes survey carried out within 4 Mb flanking the QTNs, revealed putative biological functions associated with lipid transfer and metabolism, cell wall modifications, cell cycle, and photosynthesis. Four genes encoded as *Cellulose Synthase* (*CeSa*), *Anaphase promoting complex* (*APC*/*C*), *Glucoronoxylan 4-O Methyltransferase* (*GXM*) and *HYPONASTIC LEAVES1* (*HYL1*) might be responsible for an increase in cellulose, and natural and acid detergent fiber (NDF and ADF) content in tetraploid wheat. In addition, the SNP marker RFL_Contig3228_2154 associated with the variation in stem solidness (*Q.Scsb-3B*) was validated through two molecular methods (High resolution melting; HRM and RNase H^2^-dependent PCR; rhAMP).

**Conclusions:**

The study provides new insights into the genetic basis of biomass composition traits on tetraploid wheat. The application of six ML-GWAS models on a panel of diverse wheat genotypes represents an efficient approach to dissect complex traits with low heritability such as wheat straw composition. The discovery of genes/genomic regions associated with biomass production and straw quality parameters is expected to accelerate the development of high-yielding wheat varieties useful for biofuel production.

**Supplementary Information:**

The online version contains supplementary material available at 10.1186/s12870-022-03900-6.

## Background

Wheat yield has more than doubled over the last century because of genetic improvement mainly due to the effect produced by the breeding activity on plant height and floret fertility [1, 2]. Strong selective pressure was exerted on the harvest index (expressed as ratio between the grain weight and total plant biomass), for which values of 40–50% were recorded [2]. On the contrary, the total biomass of wheat genotypes, including tetraploid wheat, remained almost unchanged, indicating that the increase in yield was associated exclusively with a different relocation of photosynthates [3].

More recently, crop residues (*i.e.,* wheat straw) are playing an increasingly important role as a source of renewable energy since modern technological processes aim at the use of inexpensive raw materials (second-generation biofuels) and are more suitable to produce bioethanol and biogas [4, 5]. Unfortunately, the complex structure of these lignocellulosic materials makes the polymers they contain (cellulose, hemicellulose, and lignin) very resistant to fungal enzymatic and chemical degradation with a low conversion efficiency, significantly reducing their use to produce biofuels [6]. Therefore, it might be appropriate to alter the chemical properties of wheat straw preserving the levels of defense against pathogens which also depends largely on the cell wall components [7, 8].

To ensure the supply of this type of biomass to the bioenergy industry, an integrated systems biology approach is needed to define the plant ideotype capable of optimizing the production of biofuels without compromising the food production of the crop. It needs to adopt a multidisciplinary approach combining plant physiology, biochemistry, molecular genetics, and genomics technologies to improve the total biomass production and optimize the composition of crop residues to the needs of the bioenergy industries [9]. Until now, the preferred strategy for modifying cell wall components in many species of bioenergetic interest was through the isolation of mutants [10] defective in the synthesis of some enzymes involved in the lignin biosynthetic pathway (brown- midrib). The use of these mutants, however, is hampered by low biomass production and low yields [11, 12].

Although numerous genomic tools are widely available for different cereal species, the complexity of the lignin and cellulose synthesis system makes the biotechnological approaches for altering the synthesis of these sugars very difficult in both bread and durum wheat. To overcome this issue, the exploration and exploitation of the genetic variability within wheat species appeared much more suitable to map the genetic determinants of the traits of interest. Genome-wide association study (GWAS) has been a routine and powerful approach for high-resolution genetic mapping of complex traits in plants [13]. Economically important traits include agronomic and yield associated traits (reviewed by [14] as well as biotic [15–17] and abiotic stress tolerance [18–21] have been mapped using GWAS in wheat. Conventionally, GWAS was performed using a single-locus mixed linear model (SL-MLM) [22]. In the last few years, multi-locus mixed linear models (ML-GWAS) have been developed, as they have higher power to detect significant marker-trait associations for complex traits than conventional SL-MLM methods [23, 24], 2018, [25–28].

ML-GWAS involve a multi-dimensional genome scan in which the effects of all markers are simultaneously estimated and does not require a multiple test correction, a statistic test that can be too conservative, especially when analyzing complex traits regulated by many genes with small effects. For these reasons, here we selected six ML-GWAS models that involve two-steps. During the first step, a single-locus GWAS method is applied to scan the entire genome, and putative QTNs are detected according to a less stringent critical value, such as *P* < 0.005 or *P* < 1/m, where m is the number of markers. During the second step, all selected putative QTNs are examined by a multi-locus GWAS model to detect true QTNs. Since markers effects are simultaneously tested in ML-GWAS models, they can represent appropriate genetic models for molecular dissection of complex traits such as those involved in biomass compositions. On this basis, the present study aimed to: i) evaluate the wheat straw composition and morphological traits through ML-GWAS using a collection of tetraploid wheat species; ii) identify novel genomic regions associated with these traits and suggest candidate genes, and iii) validate SNPs markers for marker-assisted selection.

## Results

### Phenotypic variation and correlations analysis

**Fig. 1:**
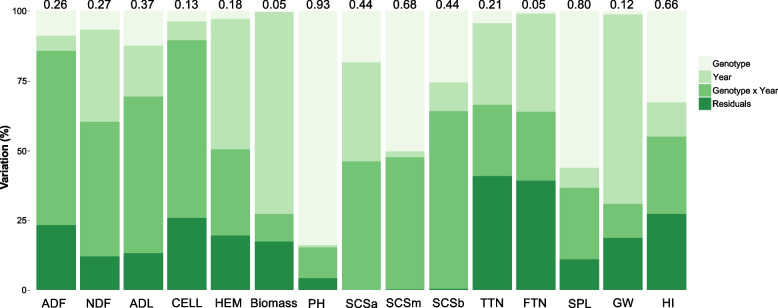
Variation component analysis with phenotypic traits measured in 185 wheat genotypes. The plot shows the percentage of variation explained by each component. The components of phenotypic variation are: i) Genotype, ii) Year, iii) interaction (Genotype x Year) and iv) residuals as percentage of the observed variation. The value on top of bars represents the broad-sense hereditability for each trait

**Fig. 2:**
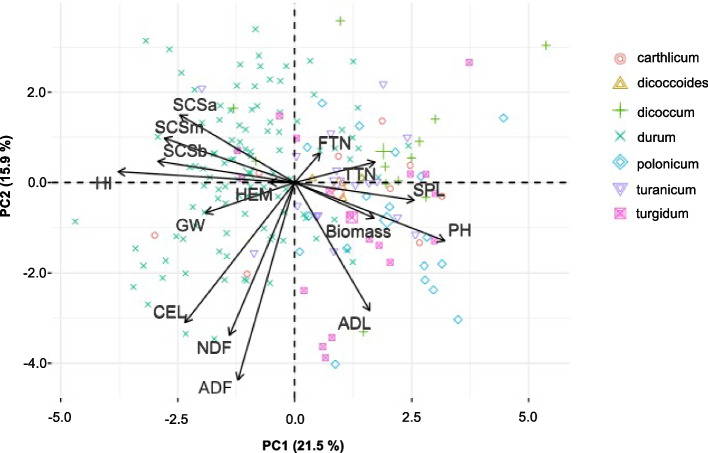
Whole phenotypic variability of the 185 wheat genotypes. Loading plot of the first (PC1) and second (PC2) principal components showing the variation for 15 traits. Based on *Triticum ssp.,* genotypes are represented by different colored symbols indicated in the legend. Trait contributions are show with arrows. The direction and distance from the center of the biplot indicate how each trait contributes to the first two components. Trait acronyms are in Supplementary Table 3

**Fig. 3:**
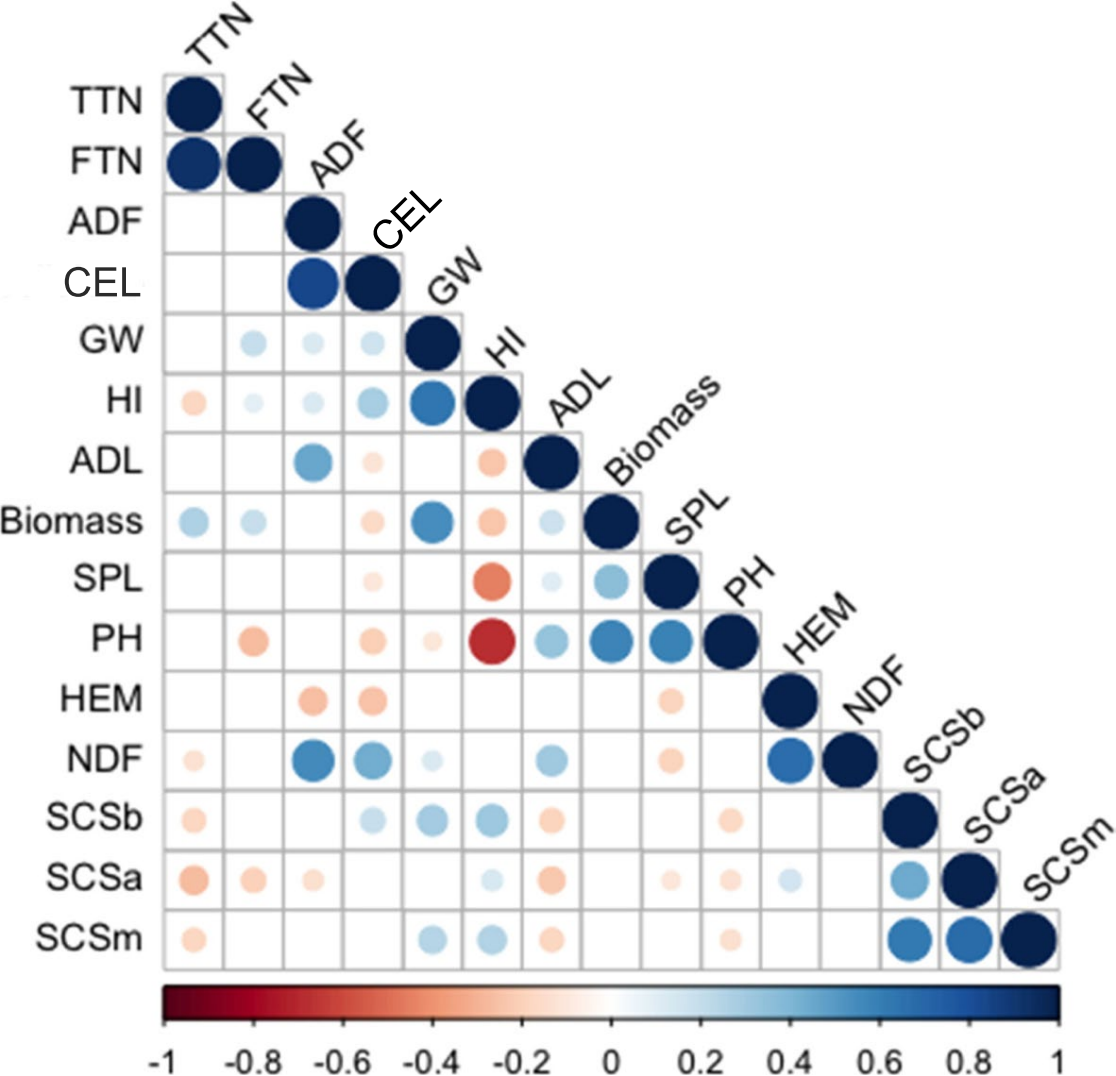
Pearson rank correlation coefficients between pairs of phenotypes. Correlation coefficients are indicated in each cell. Colored correlations are those with *P* value < 0.05 after Bonferroni correction. Color intensity is directly proportional to the coefficients. On the below side of the correlogram, the legend color shows the correlation coefficients and the corresponding colors. Trait acronyms are reported in Supplementary Table 3

The data set of three-year field trials was examined with an ANOVA to reveal: (i) genotype effect, (ii) year effect, (iii) interaction between genotype and year, and (iv) residuals (Fig. 1). Using BLUP values, the percentage of variation attributed to the genotype ranged between 11.2% and 90.1% for Biomass and PH, respectively. The year effect was generally low, except for Biomass and GW, for which it accounted for 72.1% and 67.8%, respectively. The genotype × year interaction was higher for ADF and SCSb, where it was higher than 60%. By contrast, it was lower for the remaining traits, reaching minimal values of 10.1% for Biomass. The same dataset was also used to calculate hereditability (H^2^). The H^2^ values ranged from a minimum of 0.05 for Biomass and FTN to a maximum of 0.93 for PH (Fig. 1). H^2^ values higher than 0.60 were observed for traits such as SCSm, HI, PH, and SPL, whereas values lower than 0.30 were identified for TTN, FTN, Biomass, ADF, NDF, CEL, HEM and GW. Best linear unbiased predictors (BLUPs) were then calculated for all traits and used for PCA, Pearson correlation analysis, and ML-GWAS. BLUPs distribution is reported in Supplementary Fig. 1 and in Supplementary Table 1. Differences based on *Triticum* subspecies have been observed within BLUPs distribution (Supplementary Fig. 2). For example, durum wheat accessions showed the lowest and highest BLUPs values for PH and HI, respectively. Significant differences were also observed for ADL and SPL (Supplementary Fig. 2) for durum wheat and other tetraploid subspecies. The PCA graph confirmed the variability of accessions according to the subspecies, highlighting a differentiation of the durum accessions from the rest (Fig. 2). The first five principal components (PCs) explained up to 73% of the total variance. Among them, the first two (PC1 and PC2) accounted the 37.4% of the total variation, with 21.5% and 15.9% for PC1 and PC2, respectively. Biomass and related traits such as FTN, TTN, SPL, PH, and Biomass were mainly influenced by PC1 (Fig. 2), whereas PC2 was mostly attributed to SCSa, SCSb, SCSm, NDF, ADF, and CEL. PC1 also discriminated durum wheat genotypes from other accessions of tetraploid wheat. Pearson correlation was employed to deeper understand the pairwise relationships among the traits under investigation (Fig. 3). Sixty-two significant correlation trait-pairs (*P* ≤ 0.05) were identified among all the traits. Out of all, 34 were positively correlated, while other 28 were correlated negatively. A highly positive correlation was found between FTN and TTN and, CEL and ADF (*r* = 0.96 and 0.83), whereas PH and HI together with SPL and HI showed the highest negative correlation (*r* =  − 0.67 and − 0.44). In addition, SCSa, SCSm and SCSb were all positively correlated between each other (*r* > 0.40), whereas they were negatively correlated with TTN, ADL, and PH (Fig. 3). ADL, Biomass, SPL, and PH were also negatively correlated with CEL and HI.

### SNP markers statistics, population structure and linkage disequilibrium

**Fig. 4:**
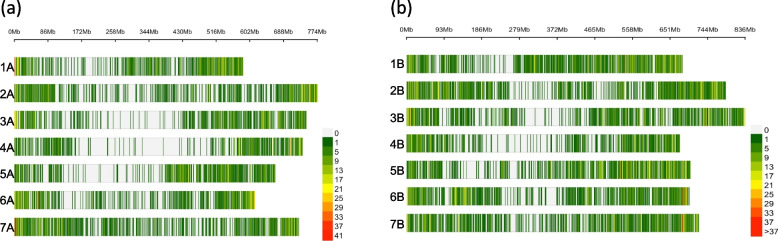
SNP density plot showing the number of SNPs within 1 Mb window size along sub genome A (a) and B (b). The horizontal axis shows the chromosome length (Mb); the different color depicts SNP density

A total of 6,090 variants were removed from the raw dataset due to missing genotype data, whereas other 49,875 variants removed due to minor allele (MAF) threshold(s), yielding a total of 20,755 filtered variants. The 20,755 SNPs included 9,199 and 11,557 on the sub-genomes A and B, respectively. The number of markers on each chromosome ranged between a minimum of 954 SNPs for chromosome 4A and a maximum of 2,075 on chromosome 2B, as shown in Fig. 4. On sub-genome A, the maximum number of SNPs were on Chr. 7A (1,687), followed by 2A (1,431) and 4A (954). By contrast, the highest number of SNPs on the sub-genome B was detected for Chr. 2B (2,075), followed by 1B (1,984) and 4B (1,031). The SNPs distribution on durum wheat chromosomes is provided in Fig. 4.

The filtered panel of 20,755 SNP markers was then used to investigate the population structure of 185 tetraploid wheat genotypes on the basis of a PCA method. The analysis indicated the existence of three distinct major clusters (Supplementary Fig. 3). The first dimension (PCA1) separated all durum wheat samples (ssp. *durum*) from the others, with the exception of few individuals belonging to ssp. *turanicum* which were included in the cluster together with durum genotypes. Durum wheat cultivars were also distributed along the PCA2 axis, separating the ancient/old varieties such as Aziziah, Timilia, Grifoni and Capeiti (quadrant IV) from the more recent ones (quadrant I), which included elite varieties mainly derived from national and international breeding programs. In addition, PCA2 further distinguished accessions belonging to ssp. *turgidum*, *turanicum* and *polonicum* from those belonging to ssp. *carthlicum*, *dicoccum* and *dicoccoides,* with some exceptions. Linkage disequilibrium (LD) decay distance at which r^2^ fell to 0.20 was ∼ 1.8 Mb, 1.6 Mb and 1.7 Mb in whole, A and B genome, respectively (Supplementary Fig. 4).

### Genome-wide association analysis

ML association mapping analysis was conducted using only the correction for kinship as it resulted the most appropriate method for the panel under study. A total of 470 significant QTNs were found associated with 15 traits (LOD ≥ 3). Out of all, 49, 64, 23, 166, 90, 68 QTNs were detected using mrMLM, pLARmEB, FASTmrEMMA, pKWmEB, FASTmrMLM and ISIS EM-BLASSO, respectively (Supplementary Fig. 5). QQ plots related to each trait are reported in Supplementary Fig. 6. Only 72 QTNs for 15 traits (two for ADL and GW, three for ADF, HEM, FTN and TTN, four for CEL, Biomass, NDF and HI, five for SCSb, six for SCSa, eight for SCSm, ten for SPL, and eleven for PH), detected by at least two methods, were declared as reliable QTNs (Table 1) and used in downstream analysis. Among these latter, BobWhite_c44947_277, associated with SPL, was detected by all methods, whereas RFL_Contig3228_2154 and wsnp_Ex_c24135_33382521, associated with SCSb and ADL, respectively, were detected by five methods (Table 1).

**Table 1:** Sixty-one QTNs identified using two or more than two multi-locus GWAS models. logarithm of the odds (LOD) and phenotypic variance explained (R^2^%) for each QTN are also reported. Trait acronym are reported in Supplementary Table 3

S. No	QTN	Trait	Marker	Allele	Physical position (bp)	LOD	R^2^ (%)	Method
1	*Q.Adf-1B*	ADF	GENE-0173_168	A/G	1B: 293,176,664	3.62–3.62	3.57–3.57	1,5
2	*Q.Adf-3B*	ADF	Kukri_c22235_1547	G/T	3B: 786,404,075	3.52–6.85	3.72–8.77	2,3,5,6
3	*Q.Adf-5A*	ADF	Kukri_rep_c101981_260	G/T	5A: 319,304,864	3.19–4.27	1.47–10.05	2,4,6
4	*Q.Adl-2B.1*	ADL	BobWhite_c30140_119	A/G	2B: 745,946,175	4.10–5.35	2.77–8.48	1,2
5	*Q.Adl-2B.2*	ADL	wsnp_Ex_c24135_33382521	A/G	2B: 702,731,805	3.04–10.79	3.90–17.42	1,2,3,4,5
6	*Q.Cel-2A*	CEL	Ku_c8927_2075	C/T	2A: 685,865,037	8.87–5.51	6.71–14.74	1,5
7	*Q.Cel-3A.1*	CEL	BobWhite_c38444_238	G/T	3A: 676,958,917	3.06–3.16	0.56–1.70	5,6
8	*Q.Cel-3A.2*	CEL	BS00048633_51	A/G	3A: 722,371,044	3.04–4.76	3.32–6.65	1,2,3
9	*Q.Cel-4A*	CEL	Excalibur_c24511_1196	A/G	4A: 607,877,731	3.30–8.16	2.90–7.73	1,4,6,5
10	*Q.Ttn-2A*	TTN	BS00068050_51	C/T	2A: 7,171,528	3.04–4.36	0.29–4.81	1,2,4
11	*Q.Ttn-2B*	TTN	Tdurum_contig74936_456	A/G	2B: 79,054,183	3.29–6.82	0.00–7.87	4,6
12	*Q.Ttn-7A*	TTN	Kukri_c18148_913	C/T	7A: 715,952,715	3.30–4-84	2.32–8.80	1,3
13	*Q.Hi-4A*	HI	Tdurum_contig42638_383	A/C	4A: 727,573,605	3.18–9.11	1.22–20.94	1,2,6
14	*Q.Hi-7A.1*	HI	BS00044593_51	A/G	7A: 123,464,462	5.41–7.82	0.12–6.04	5,6
15	*Q.Hi-7A.2*	HI	RFL_Contig2834_890	C/T	7A: 714,390,256	3.33–5.73	1.60–5.05	2,5,6
16	*Q.HI-7B*	HI	RAC875_c48671_172	A/G	7B: 719,682,848	3.17–3.87	1.10–1.59	5,6
17	*Q.Biomass-2B.1*	BIOMASS	Excalibur_c34937_710	G/T	2B: 6,711,112	3.42–6.85	8.49–14.79	1,5,6
18	*Q.Biomass-2B.2*	BIOMASS	RAC875_c31214_58	C/T	2B: 770,956,440	4.30–5.48	5.99–7.77	1,5,6
19	*Q.Biomass-4B*	BIOMASS	RAC875_c25045_637	C/T	4B: 548,976,483	3.21–3.98	6.32–10.91	1,2,5,6
20	*Q.Biomass-5A*	BIOMASS	wsnp_Ex_c1138_2185522	A/G	5A: 533,045,040	3.13–5.67	0.00–8.00	1,6
21	*Q.Gw-4A*	GW	Ra_c1897_2401	C/T	4A: 702,329,094	3.73–6.89	4.82–11.00	1,2,3,6
22	*Q.Gw-7B*	GW	BS00068071_51	A/G	7B: 173,518,366	3.28–3.56	0.00–8.47	2,6
23	*Q.Ndf-2A*	NDF	Tdurum_contig31185_456	C/T	2A: 741,540,226	3.78–4.30	3.81–5.58	2,3,6
24	*Q.Ndf-4B.1*	NDF	BS00024110_51	G/T	4B: 89,594,936	3.34–4.15	0.00–10.34	2,5,6
25	*Q.Ndf-4B.2*	NDF	BS00030571_51	A/G	4B: 606,290,375	3.69–7.23	3.67–8.11	2,5,6
26	*Q.Ndf-7A*	NDF	BobWhite_c25527_980	C/T	7A: 709,196,631	3.38–4.46	0.00–1.01	2,5
27	*Q.Scsa-1A.1*	SCSa	BS00110627_51	G/T	1A: 527,767,957	4.09–6.14	6.51–7.76	3,5
28	*Q.Scsa-1A.2*	SCSa	Tdurum_contig51833_439	A/G	1A: 537,137,625	3.62–7.12	10.68–13.16	1,4,6
29	*Q.Scsa-2A*	SCSa	GENE-1381_110	G/T	2A: 685,560,391	3.25–4.02	1.52–4.81	1,4,6
30	*Q.Scsa-3A*	SCSa	RAC875_c3084_415	C/T	3A: 728,749,058	4.53–6.16	12.00–15.55	1,6
31	*Q.Scsa-3B*	SCSa	BS00059887_51	A/G	3B: 748,418,401	3.24–5.67	0.00–9.96	1,2
32	*Q.Scsa-6A*	SCSa	BS00068218_51	C/T	6A: 599,877,040	3.58–5.09	2.31–6.24	1,2,4
33	*Q.Scsm-1A*	SCSm	wsnp_Ex_c4186_7560575	C/T	1A: 584,664,979	3.48–3.96	2.70–3.54	2,5
34	*Q.Scsm-2B*	SCSm	BS00025649_51	A/C	2B: 704,739,952	3.33–4.84	5.58–6.59	1,2
35	*Q.Scsm-3B*	SCSm	RAC875_c63132_298	C/T	3B: 6,886,640	4.11–4.51	7.62–11.95	4,6
36	*Q.Scsm-4A*	SCSm	RAC875_c18732_193	G/T	4A: 380,699,504	4.11–9.09	5.74–12.14	1,2,5,6
37	*Q.Scsm-6B.1*	SCSm	BS00011192_51	A/G	6B: 681,643,033	3.34–3.64	5.12–7.39	1,6
38	*Q.Scsm-6B.2*	SCSm	RAC875_c49329_81	C/T	6B: 684,859,721	3.36–4.67	5.48–7.75	4,5
39	*Q.Scsm-6B.3*	SCSm	wsnp_BF293311B_Ta_2_3	A/C	6B: 439,365,045	4.43–4.73	7.23–10.44	1,2
40	*Q.Scsm-6B.4*	SCSm	Excalibur_c64119_578	C/T	6B: 12,368,785	5.22–5.83	7.97–10.88	1,2,4,5
41	*Q.Scsb-2B*	SCSb	Excalibur_c10068_1276	A/G	2B: 743,736,881	4.92–9-32	8.55–19.14	1,2,3
42	*Q.Scsb-3B*	SCSb	RFL_Contig3228_2154	C/T	3B: 21,113,077	3.81–8.78	8.96–13.11	1,2,3,4,6
43	*Q.Scsb-4A*	SCSb	Tdurum_contig15260_650	A/G	4A: 721,237,375	3.05–3.11	5.21–6.06	1,2
44	*Q.Scsb-6A.1*	SCSb	wsnp_Ku_c26784_36748247	C/T	6A: 50,651,609	3.07–4,07	1.90–2.20	4,6
45	*Q.Scsb-6A.2*	SCSb	BS00036878_51	A/G	6A: 452,642,718	3.43–4.95	0.00–1.65	2,4,6
46	*Q.Hem-4B*	HEM	BS00003765_51	C/T	4B: 601,823,368	3.78–5.03	2.13–4.76	3,5
47	*Q.Hem-5A*	HEM	Ra_c19494_275	C/T	5A: 625,718,974	4.38–7.19	4.53–3.34	5,6
48	*Q.Hem-6B*	HEM	RAC875_c47035_70	A/G	6B: 442,960,966	3.14–5.69	0.00–7.19	4,5,6
49	*Q.Spl-1A.1*	SPL	D_GBUVHFX01API9H_416	A/G	1A: 560,604,148	7.13–12.54	5.72–11.96	1,2
50	*Q.Spl-1A.2*	SPL	BobWhite_c44947_277	C/T	1A: 580,573,915	3.08–7.44	2.43–6.39	1,2,3,4,5,6
51	*Q.Spl-3B*	SPL	Tdurum_contig12087_190	A/C	3B: 736,504,198	5.85–7.87	10.33–12.68	1,3
52	*Q.Spl-4A.1*	SPL	RAC875_c34515_343	A/G	4A: 617,606,418	3.84–5.41	1.87–4.76	1,4
53	*Q.Spl-4A.2*	SPL	Tdurum_contig47858_908	A/G	4A: 618,975,358	5.04–8.08	2.36–3.60	4,6,5
54	*Q.Spl-4B.1*	SPL	Ex_c14614_433	A/G	4B: 114,625,178	4.51–8.48	4.86–7.91	3,6
55	*Q.Spl-4B.2*	SPL	Excalibur_c12937_544	A/G	4B: 654,334,669	4.97–9.87	4.73–9.25	1,6
56	*Q.Spl-4B.3*	SPL	Excalibur_rep_c70127_360	A/G	4B: 560,440,302	3.22–11.65	0.00–9.03	1,2,4
57	*Q.Spl-6A*	SPL	GENE-3665_61	A/G	6A: 36,191,483	3.41–4.04	1.86–1.86	2,6
58	*Q.Spl-7A*	SPL	wsnp_Ku_c792_1635653	C/T	7A: 9,266,099	4.54–4.73	3.04–3.55	5,6
59	*Q.Ftn-2B*	FTN	Excalibur_c27769_1089	C/T	2B: 135,830,179	3.04–5.82	0.37–1.11	2,6
60	*Q.Ftn-5A*	FTN	BS00000365_51	A/G	5A: 501,011,928	5.72–11.41	3,12–5.10	2,6
61	*Q.Ftn-6A*	FTN	Kukri_c45702_439	A/G	6A: 602,501,261	4.37–6.03	0.96–2.12	2,6
62	*Q.Ph-1A*	PH	Excalibur_c6255_1119	T/C	1A: 152,822,081	12.02–16.10	12.45–19.78	1,5
63	*Q.Ph-2A.1*	PH	BS00089497_51	G/A	2A: 688,361,342	5.30–6.54	2.09–2.55	1,2,3,4
64	*Q.Ph-2A.2*	PH	CAP12_rep_c6956_169	T/C	2A: 691,749,827	4.32–7.61	1.20–2.04	2,3
65	*Q.Ph-2A.3*	PH	BS00107804_51	C/T	2A: 701,377,125	4.48–5.36	1.69–2.89	4,6
66	*Q.Ph-3A*	PH	wsnp_Ex_c20250_29303152	G/T	3A: 687,446,416	3.95–14.09	2.30.7.37	3,6
67	*Q.Ph-4B.1*	PH	BS00023766_51	T/C	4B: 30,278,520	4.26–7.99	2.10–4.18	3,6
68	*Q.Ph-4B.2*	PH	Tdurum_contig63670_287	G/A	4B: 35,030,729	4.29–17.11	3.79–9.61	1,2,4,5
69	*Q.Ph-5B.1*	PH	RAC875_c39204_91	C/T	5B: 23,191,553	5.17–12.82	2.76–6.47	1,4,5
70	*Q.Ph-5B.2*	PH	BS00068200_51	T/C	5B: 510,428,487	3.89–6.75	1.67–2.84	2,3
71	*Q.Ph-7A*	PH	RAC875_c28585_156	C/T	7A: 16,934,067	4.21–4.72	1.51–1.87	2,3
72	*Q.Ph-7B*	PH	BobWhite_rep_c49050_1890	A/G	7B: 622,291,386	3.99–9.81	0.93–2.73	2,4,6

### QTNs for biomass and chemical composition

Twenty QTNs were identified for Biomass and five related traits (ADF, ADL, NDF, CEL, and HEM) by at least two different models and then considered as reliable (Table 1). They were distributed on ten chromosomes: 1B, 2A, 2B, 3A, 4A, 4B, 5A, 6B, 7A, 7B. Three reliable QTNs were identified for ADF. Among these, *Q.Adf-5A* was annotated as major QTN (R^2^ ≥ 10 at least in one method and LOD values ranging between 3.19–4.27), whereas the other two (*Q.Adf-1B and Q.Adf-3B*) as minor. Two QTNs on chromosome 2B, (one major *Q.Adl-2B.2* and one minor, *Q.Adl-2B.1*) were instead identified for ADL. Four reliable QTNs were identified for CEL; *Q.Cel-2A* was considered major as it explained the highest phenotypic variance (6.71–14.74%), whereas the remaining three were declared as minor since their R^2^ values were < 10%. Four QTNs were also identified for Biomass and NDF. Two QTNs for Biomass (*Q.Biomass-2B.1* and *Q.Biomass-4B*) and one for NDF (*Q.Ndf-4B.1*) were annotated as major, whereas the others were as minor.

### QTNs for morphological traits

Fifty-two reliable QTNs were significantly associated with nine traits morphological traits, and they were distributed on all chromosomes except for chr. 1B (Table 1). The highest number of QTNs were identified for PH (11) whereas the lowest number was found for GW (2). Among the QTNs identified for PH, only *Q.Ph-1A* was considered major (R = 19.78%), whereas among the QTNs identified for SPL, two (*Q.Spl-1A.1* and *Q.Spl-3B*) were considered major. In particular, *Q.Spl-1A.1* explained phenotypic variation ranging between 5.72% and 11.96% and it showed the highest LOD values (7.13–12.54). Eight reliable QTNs were associated with SCSm, of which four were major (*Q.Scsm-3B*, *Q.Scsm-4A*, *Q.Scsm-6B.3* and *Q.Scsm-6B.4*). Among these latter, *Q.Scsm-4A* explained the highest phenotypic variance (12.14%) with LOD values ranging between 4.11–9.09. Six and five reliable QTNs were associated with SCSa and SCSb, respectively. In particular, two QTNs for SCSa (*Q.Scsa-1A.2* and *Q.Scsa-3A*) and two for SCSb (*Q.Scsb-2B* and *Q.Scsb-3B*) were major. Four QTNs were instead identified for HI. Out of all, *Q.Hi-4A* was declared as major, since it explained up to 20.94% of the phenotypic variation. Three QTNs for TTN and FTN were also identified, but they explained a phenotypic variation of < 10%. Finally, two QTNs were identified for GW, of which one was major (*Q.Gw-4A*).

### Allelic effect of major QTNs on biomass traits

**Fig. 5:**
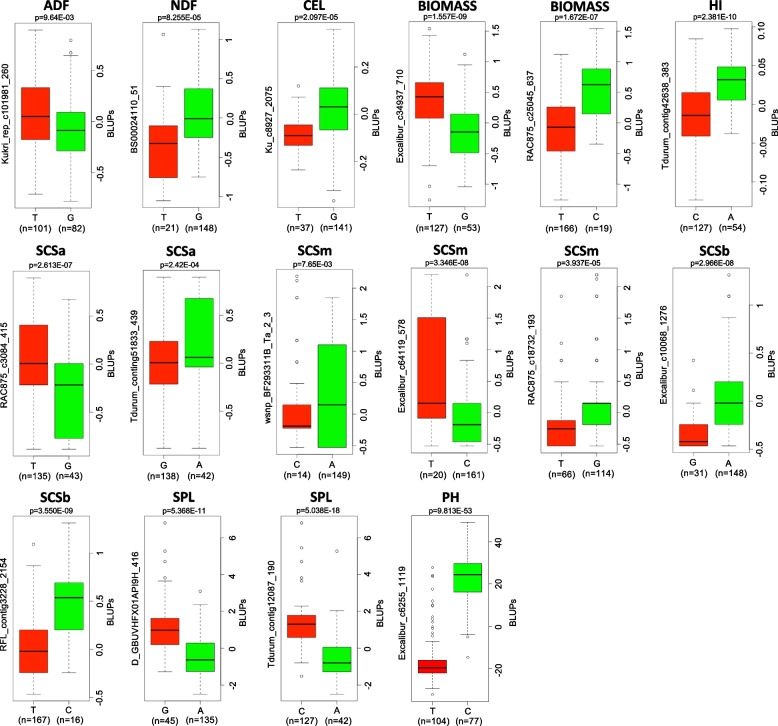
Boxplot for 15 reliable QTNs with significant effects (*P* < 0.01) on corresponding traits. For each QTNs, the germplasm lines were divided into two groups according to superior and inferior allele type. The X-axis represents the two alleles for each QTNs, while the Y-axis corresponds to BLUP values

The major QTNs (R^2^ ≥ 10) were also tested using t-test (*P* ≤ 0.01) (Fig. 5). We divided the population into two groups according to allelic profile to test whether the mean BLUP values of the two groups were significantly different. In total, 16 QTNs had a significant effect on nine traits (Fig. 5). Among these, the highest number of QTNs (three) were significant for SCSm (*Q.Scsm-4A*, *Q.Scsm-6B.3*, *Q.Scsm-6B.4*). Two QTNs were significant for SCSa (*Q.Scsa-1A.2*, *Q.Scsa-3A*), SCSb (*Q.Scsb-2B*, *Q.Scsb-3B*), SPL (*Q.Spl-1A.1*, *Q.Spl-3B*) and Biomass (*Q.Biomass-2B.1*, *Q.Biomass-4B*). One QTN showed significant effect on ADF (*Q.Adf-5A*), CEL (*Q.Cel-2A*), HI (*Q.Hi-4A*), NDF (*Q.Ndf-4B.1*), and PH (*Q.Ph-1A*).

### Identification of putative candidate genes associated with major QTNs

Genomic regions (± 1.8 Mb) surrounding the sixteen major QTNs with allelic effects were investigated (Table 2). Several genes modulating lipid, carbohydrate, and starch-sucrose metabolisms, as well as genes involved in photosynthetic processes and secondary metabolites production, were annotated within QTNs. For example, three synthases, *Cellulose synthase* (*CESA*), *Sucrose Synthase 6* (*SuSy*) and *Glucan synthase-like 4* (*GSL4*), along with two transferases *Beta-fructofuranosidase* (*CIN4*) and *Sterol 3-beta-glucosyltransferase*, all involved in sucrose and starch metabolism, were found associated with SCSa, SPL and SCSm, respectively.

**Table 2:** Candidate genes around the reliable QTNs and their functional annotation. The durum (Svevo) gene ID along with direct orthologs in rice is also reported

QTN	Trait	Marker	Gene ID	Gene Description	Rice Ortholog	Rice gene name
*Q.Adf-5A*	ADF	Kukri_rep_c101981_260	TRITD5Av1G111780	Non-specific lipid-transfer protein (LTP)	Os12g0115500	OsLTPL14/OsLTP1.20
			TRITD5Av1G111860	Anthocyanidin 3-O-glucosyltransferase 2 G (UFGT)	NA	NA
			TRITD5Av1G112460	Lipid transfer protein (LTP)	Os12g0112401	NA
			TRITD5Av1G112990	Double-stranded RNA-binding protein 3 (HYL1)	NA	NA
			TRITD5Av1G112500	Peroxidase (PRX)	Os12g0112000	OsPRX1
*Q.Cel-2A*	CEL	Ku_c8927_2075	TRITD2Av1G252600	Anthocyanin 3'-O-beta-glucosyltransferase (3'GT)	NA	NA
			TRITD2Av1G253060	Anaphase promoting complex (APC/C)	Os04g0599800	OsCDC20
			TRITD2Av1G253600	Anthocyanin 5-aromatic acyltransferase (3AT1)	NA	NA
*Q.Hi-4A*	HI	Tdurum_contig42638_383	TRITD4Av1G259790	Scarecrow transcription factor family protein	Os06g0105350	OsGRAS31
			TRITD4Av1G259630	Acetyltransferase component of pyruvate dehydrogenase complex	Os06g0105400	NA
			TRITD4Av1G259520	Mitochondrial pyruvate carrier	Os07g0449100	NA
*Q.Biomass-2B.1*	BIOMASS	Excalibur_c34937_710	TRITD2Bv1G002680	Terpene synthase (TPS)	NA	NA
			TRITD2Bv1G002820	Pyruvate dehydrogenase E1 component subunit alpha	Os04g0119400	NA
			TRITD2Bv1G002950	Gibberellin-regulated protein 1 (GASA)	NA	NA
			TRITD2Bv1G003120	WRKY transcription factor	Os11g0490900	OsWRKY72
			TRITD2Bv1G003210	Fatty acyl-CoA reductase 1 G (FAR)	NA	NA
			TRITD2Bv1G003590	Sterol 3-beta-glucosyltransferase (UGT80B1)	Os04g0131900	NA
			TRITD2Bv1G004160	Fructokinase-2 (FRK)	Os03g0602600	OsHSA1
			TRITD2Bv1G003650	Cell differentiation protein RCD1	Os02g0301700	NA
*Q.Biomass-4B*	BIOMASS	RAC875_c25045_637	TRITD4Bv1G160470	4-coumarate–CoA ligase family protein (4CL)	Os03g0152400	NA
			TRITD4Bv1G160730	Flavin-containing monooxygenase	NA	NA
			TRITD4Bv1G161530	Ripening related protein family	Os10g0490666	NA
			TRITD4Bv1G160440	Peroxidase (PRX)	Os03g0152300	OsPRX36
*Q.Ndf-4B.1*	NDF	BS00024110_51	TRITD4Bv1G031160	Glucuronoxylan 4-O-methyltransferase	Os11g0242600	NA
			TRITD4Bv1G031370	Chlorophyll a-b binding protein, chloroplastic	Os11g0242800	OsLHCB5
			TRITD4Bv1G030990	ethylene-responsive transcription factor	Os11g0242300	OsERF19
			TRITD4Bv1G031510	Zinc finger-homeodomain protein 1	Os11g0243300	OsZHD4
*Q.Scsa-1A.2*	SCSa	Tdurum_contig51833_439	TRITD1Av1G205480	Late embryogenesis abundant protein	Os05g0542500	OsLEA3/OsLEA19
			TRITD1Av1G205500	StAR-related lipid transfer protein	Os02g0468400	NA
			TRITD1Av1G205630	Glucuronoxylan 4-O-methyltransferase	NA	NA
			TRITD1Av1G205550	Pectin lyase-like superfamily protein	Os05g0542800	OsPGL9
			TRITD1Av1G205720	MYB transcription factor	NA	NA
			TRITD1Av1G206070	Transcription factor GTE1	Os06g0138000	NA
			TRITD1Av1G206530	Chlorophyll a-b binding protein, chloroplastic	NA	NA
*Q.Scsa-3A*	SCSa	RAC875_c3084_415	TRITD3Av1G277350	Abscisic stress ripening	Os01g0959100	OsASR5/OsASR1
			TRITD3Av1G277360	Peroxidase	Os01g0963000	OsPRX22
			TRITD3Av1G277710	Transcription factor MYB	Os01g0142500	NA
			TRITD3Av1G278300	Cellulose synthase G	NA	NA
			TRITD3Av1G278410	GDSL esterase/lipase	Os04g0650200	OsGELP59
			TRITD3Av1G278770	Photosystem II D2 protein	NA	NA
			TRITD3Av1G279110	Beta-fructofuranosidase, insoluble protein	Os01g0966700	OsCIN4
			TRITD3Av1G279470	Invertase/pectin methylesterase inhibitor family protein	NA	NA
*Q.Scsm-4A*	SCSm	RAC875_c18732_193	TRITD4Av1G122990	3-oxoacyl-[acyl-carrier-protein] synthase	NA	NA
			TRITD4Av1G123370	ADP-L-glycero-D-manno-heptose-6-epimerase G	NA	NA
			TRITD4Av1G123360	WD40 repeat-like protein	Os11g0660300	OsRBP10
*Q.Scsm-6B.3*	SCSm	wsnp_BF293311B_Ta_2_3	TRITD6Bv1G134450	Hexosyltransferase	Os02g0624400	OsPGSIP-B1
			TRITD6Bv1G134410	glucan synthase-like 4 G	NA	NA
			TRITD6Bv1G134360	MYB	Os02g0624300	OsMYB30
			TRITD6Bv1G134250	LIGHT-DEPENDENT SHORT HYPOCOTYLS-like protein	Os02g0623400	OsG1L3
			TRITD6Bv1G134270	Glycolipid transfer protein domain-containing protein	Os02g0622400	NA
*Q.Scsm-6B.4*	SCSm	Excalibur_c64119_578	TRITD6Bv1G003860	Chalcone synthase	Os10g0168500	OsPKS19
			TRITD6Bv1G004310	SKP1-like protein	Os02g0101600	NA
			TRITD6Bv1G004640	Aspartate aminotransferase	Os02g0236000	NA
			TRITD6Bv1G003910	B3 domain-containing protein Os03g0164300	NA	NA
*Q.Scsb-2B*	SCSb	Excalibur_c10068_1276	TRITD2Bv1G246800	Lipid transfer protein (LTP)	NA	NA
			TRITD2Bv1G247690	Flowering Locus T-like protein, putative (FTL)	Os12g0232300	OsFTL7
			TRITD2Bv1G248090	Sulfotransferase	Os09g0256100	NA
			TRITD2Bv1G248160	Cyanidin-3-O-glucoside 2-O-glucuronosyltransferase G (UGAT)	NA	NA
			TRITD2Bv1G248240	basic helix-loop-helix (bHLH) DNA-binding superfamily protein	Os04g0631600	OsbHLH068
*Q.Scsb-3B*	SCSb	RFL_Contig3228_2154	TRITD3Bv1G009930	Mannose-6-phosphate isomerase (PMI1)	Os01g0127900	NA
			TRITD3Bv1G009950	MYB transcription factor	Os01g0128000	NA
			TRITD3Bv1G010560	Alpha-xylosidase G (XLY1)	NA	NA
			TRITD3Bv1G010090	Anthocyanin 5-aromatic acyltransferase-like (F511_15381)	Os06g0145400	NA
			TRITD3Bv1G009370	Dihydroflavonol-4-reductase	Os01g0127500	NA
*Q.Spl-1A.1*	SPL	D_GBUVHFX01API9H_416	TRITD1Av1G216220	Cyclin-D1-binding protein 1	Os05g0554500	NA
			TRITD1Av1G216250	Gibberellin-regulated family protein	Os10g0115550	NA
			TRITD1Av1G217110	Hexosyltransferase	Os05g0552200	NA
			TRITD1Av1G217210	sucrose synthase 6 (SUSY)	NA	NA
			TRITD1Av1G218140	Lipid phosphate phosphatase-like protein	Os05g0549900	NA
			TRITD1Av1G218210	Homeobox protein, putative	Os02g0565600	Oshox7
*Q.Spl-3B*	SPL	Tdurum_contig12087_190	TRITD3Bv1G241180	Transaldolase (TAL)	Os01g0926300	NA
			TRITD3Bv1G241450	basic helix-loop-helix (bHLH) DNA-binding superfamily protein	Os11g0634700	OsbHLH132
			TRITD3Bv1G241970	Auxin response factor	Os01g0927600	OsARF2/OsARF4
			TRITD3Bv1G242050	Transcription factor Inducer of CBF expression 1	Os01g0928000	OsbHLH001
			TRITD3Bv1G242450	Transcription factor Sox-9 G	Os01g0928700	OsLCB2a2

Similarly, three peroxidases (*PRX1*, *PRX22*, and *PRX36*), known to be involved in the phenylpropanoid-lignin pathway were found within QTNs associated with ADF, Biomass, and SCSa. In addition to these latter, *4-coumarate-CoA ligase* (*4CL*) and *Hexosyltransferase*, both belonging to the same pathway, were also found. Genes involved in hemicellulose biosynthesis were also identified among QTNs. For example, a *Glucuronoxylan 4-O-methyltransferase* involved in the modification of one of the principal components present in the secondary cell walls of plants (hemicellulose 4-O-methyl glucuronoxylan) was associated with NDF and SCSa. Interestingly, *HYPONASTIC LEAVES 1* (*HYL1*), a gene encoding a nuclear double-stranded RNA-binding protein with a role in miRNA biogenesis was found associated with ADF. Transcription factors belonging to *Ap2-like ethylene-responsive* (*AP2/ERF*), Ethylene-responsive (*ERF*), *WRKY,* and *MYB* were also annotated. Among them, a scarecrow transcription factor like *OsGRAS31*, a *WRKY* transcription factor similar to *OsWRKY72*, a Zinc finger-homeodomain protein 1 similar to *OsZHD4,* and a *MYB* similar to *OsMYB30* were associated with HI, Biomass, NDF, and SCSm, respectively.

### Marker validation through molecular methods

**Fig. 6:**
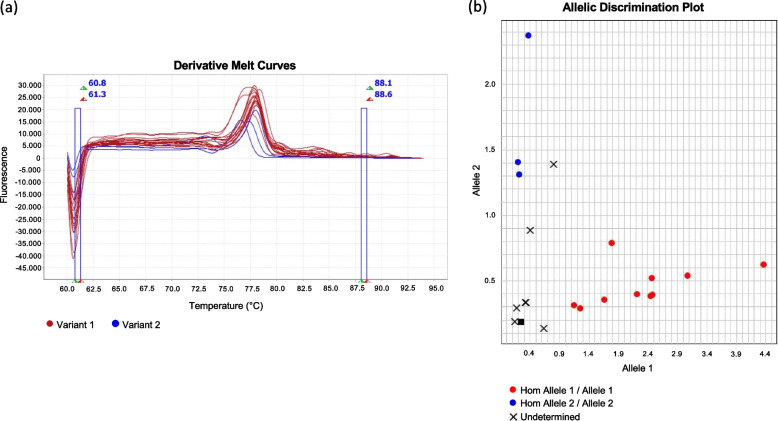
Validation of RFL_Contig3228_2154 on thirty-four genotypes using two approaches. a) Melting temperatures (Tm) from HRM analysis and b) allelic discriminations plot from rhAmp assay. Each dot represents a genotype, while the allele state (homozygous for the reference allele, homozygous for the alternate allele) are labeled with different colors. Variant 1 mean homozygous allele 1 / allele 1, whereas variant 2 refer to homozygous allele 2 / allele 2

Based on the allelic effects of the 16 reliable QTNs, one marker (*Q.Scsb-3B*, RFL_Contig3228_2154) associated with the understudied trait SCSb, was selected and validated on 34 accession included in the panel under study, using two different molecular methods (HRM and rhAMP). The marker RFL_Contig3228_2154 was able to distinguish genotypes with a strong contrasting phenotype based on their allele (Fig. 6, Supplementary Table 2), since the homozygous “AA” and “aa” profiles were associated with low and high values of SCSb, respectively. Both HRM and rhAmp analysis showed that most accessions had the allelic profile “AA”, whereas six showed homozygous “aa” genotype.

## Discussion

Wheat straw is an attractive substrate for second-generation biofuel production because it will complement and augment wheat production rather than competition with food production. Whilst many wheat varieties were developed to optimize yield and grain quality for human and animal consumption, little emphasis was given to developing the non-food components for biorefining purposes. Probably also because standard chemical analysis of large numbers of different samples was expensive and time consuming to be used in breeding programs.

A large variability was detected in the panel selected in this study, confirmed what was previously observed in the large tetraploid wheat germplasm by Laidò et al. [29] and Taranto et al. [30]. The great genetic diversity reflected the evolutionary history of tetraploid wheats. Indeed, the wild and domesticated accessions were separated from the durum wheat cultivars. These latter were spread on the PCA axes mainly based on the year of release [29]. Indeed, the ancient/old varieties clustered closed to ssp. *turgidum*, *turanicum* and *polonicum*, while the modern cultivars were spread separately from the other samples. This large genetic variability of elite cultivars may be explained by the fact that they derived from national and international breeding programs developed during the last thirty years [13].

On the contrary, the variation in the chemical composition of the biomass and related traits did not always reflect the large genetic diversity, confirming the previous observations conducted by Joshi et al. [31] and Blümmel et al. [32] on wheat straw in South Asia. Indeed, a moderate variation was observed comparing the phenotypic distribution among different wheat subspecies. Nevertheless, robust QTNs and genotypes carrying superior straw traits were identified, probably due to the sensitivity of the ML-GWAS approach. ML-GWAS models, the FASTmrMLM method was relatively faster compared to other models, as also reported by Chaurasia et al. [25]. The ISIS EMBLASSO detected the highest number of significant associations, whereas the lowest number was found with mrMLM.

Plant height plays a crucial role in biomass accumulation and grain yield and all six-multi-locus models identified QTNs associated with this well-studied trait on chromosomes 1A, 2A, 3A, 4B, 5B, 7A, and 7B, consistent with QTLs reported in previous studies [33–36]. Two of them were localized ~ 9 kb far from the main *Rht-B1* gene controlling PH trait on chromosome 4B [37], in the same genomic region where a QTL for the same trait was identified by Vitale et al. [38].

Unlike other cereal species such as maize, rice, and barley, in which numerous studies were conducted with the aim of mapping QTLs related to the straw composition [39–41], in wheat the studies were scarce, often referred to a limited number of genotypes. Malik et al. [42], searching for significant SNP markers associated to quality parameters of wheat straw, identified marker-trait associations (MTA) on chromosomes 1A, 1B, 4A, 4B, and 6A for glucose, xylose and arabinose, all traits crucial for increasing sugar release for bioethanol production.

(Supplementary Table 4). We found a marker on chromosome 4A (Excalibur_c24511_1196) associated with CEL (*Q.Cel-4A*), located 22 Mb far from the MTA for Arabinose (GENE-1756_115) identified by Malik et al. [42].

(Supplementary Table 4). By contrast, although we found QTNs for ADF, NDF, and Biomass on the same chromosomes detected by Malik et al. [42], our markers were located at least 100 Mb far, making it difficult to validate with our findings. Comparing the results with the QTLs known in the literature with the regions identified in our study, reliable QTNs on chromosomes 2A, 2B, 4B, 4A, and 5A (*Q.Biomass-2B.2*, *Q.Biomass-4B*, *Q.Hi-4A*, *Q.Adf-5A*, *Q.Cel-2A,* and *Q.Ndf-4B.1*) were coincident with previously reported QTLs for biomass accumulation [43], HI [44], grain yield [36, 45] and heading date [45].

(Supplementary Table 4). Additional QTNs, associated with biomass composition on chromosomes 3B (*Q.Adf-3B*), 2B (*Q.Adl-2B.2*), 4A (*Q.Cel-4A*), 4B (*Q.Ndf-4B.2*), and 5A (*Q.Hem-5A*), were coincident with QTLs previously identified for grain yield [45], phenolic acid content [46], biomass [36, 47], shoot dry weight [48] and heading date [47, 49], respectively. Recently, Joshi et al. [31] carried out a GWAS on 287 spring wheat lines for mapping straw fodder quality trait and identified associations for ADF, ADL, and NDF on chromosomes 1A, 2B, 3A, 5A and 5B. In our work, we found a QTN on chromosome 2B (*Q.Adl-2B.1*) which is located 21 Mb far from the MTA (Excalibur_c49875_479 on chromosome 2B) described by the authors for the same trait (ADL) (Supplementary Table 4). By contrast, no chromosomal region overlapping with our results have been found for the remaining traits. The fact that in these regions were mapped loci associated with several biomass-related traits makes them an interesting source of allelic variation to modulate their phenotypic expression. Three associations identified in this study for the SPL on chromosomes 1A (*Q.Spl-1A.1*, *Q.Spl-1A.2*) and 3B (*Q.Spl-3B*) agreed with the QTLs previously identified by Graziani et al. [50] and Maccaferri et al. [48] for the number of spikes per square meter and total root number, respectively.

Among the agronomic traits analyzed, stem solidness was also considered. Usually, the morphological features of solid stemmed wheat suggested that it could be highly resistant to lodging [51]. In addition, it was known that solid stemmed wheat varieties have increased resistance to damage from sawfly larvae, as the presence of solid pith impedes larval growth and migration [52]. In fact, wheat stem sawfly (WSS) resistant varieties with pith-filled solid stems have been selected in North America and in central Europe to help control WSS since the 1950s. There were several studies conducted to identify the genetic basis of stem solidness whereas more limited were the studies exploring the differences in the biochemical compositions between hollow- and solid-stemmed varieties. Recently, Nilsen et al. [53] demonstrated that copy number variation of *TdDof*, a gene encoding a putative DNA binding with one finger protein, affected the stem solidness trait in wheat at the *SSt1* locus on chromosome 3BL. More recent genetic studies have identified a second allele at the *Qss.msub-3BL* locus contributing to stem solidness in durum wheat. This allele was first identified in the cultivar Conan and was designated *Qss.msub-3BL.c* [1, 54]. The *Qss.msub-3BL.c* conferred a solid-stem phenotype at the early stage of stem elongation, differently from the phenotype conferred by the Rescue-derived *Qss.msub-3BL.b* allele and, was lost later in stem elongation and maturation. Given that in this study, the scoring for stem solidness was carried out at harvest time, it could be the reason why no associations with the *SSt1* locus on chromosome 3B, were found. In addition, minor QTLs were also identified on chromosomes 2A, 2D, 4A, and 5A that were found to synergistically enhance expression of *SSt1* to increase stem-solidness [55]. These previous results supported the SNPs associations found in the present study for stem solidness (SCS) at three levels of the culm (basal, medium and apical) on chromosomes 1A, 2B, 3A, 3B, 4A, and 6B. Unfortunately, the three regions mapped on 3B (*Q.Scsb-3B*, *Q.Scsa-3B* and *Q.Scsm-3B*) did not coincide with the region of the *SSt1* locus [55] whereas, they coincided with QTLs previously mapped for disease resistance traits as yellow rust resistance [56] and fusarium head blight resistance [57]. Similarly, the other QTNs identified for the SCS traits on the other chromosomes overlapped with QTLs previously mapped for resistance diseases such as *Q.Scsm-4A*, *Q.Scsb-2B and* Q.Scsm-6B.3 for leaf, yellow and stem rust [58], Liu et al. 2017b; [59] suggesting their potential involvement in other genetic resistance mechanisms in addition to the well-known resistance to WSS. Most durum wheat accessions do not possess the solid-stem *Qss.msub-3BL.b* allele for stem solidness and have been traditionally classified as hollow-stemmed. However, hollow-stem durum wheat typically has more resistance to WSS than hollow-stem hexaploid wheat [60]. Therefore, despite several studies aiming to map the loci responsible for the solid stem phenotype, the underlying molecular mechanisms contributing to this key trait remain elusive. The validated SNP marker (RFL_Contig3228_2154) associated to SCSb in the present work was previously related to different trait such as grain weight and gluten component (HMW-GS, and LMW-GS) [13, 61]. Now, this marker can be used for MAS to track differences in SCSb in tetraploid wheat accessions.

### Candidate genes surveying revealed genes involved in lipid metabolism, cell wall modifications and cell cycle

In our work, we found different classes of candidate genes in QTNs/genomic regions. For example, genes involved in the synthesis of principle components present in the secondary walls of eudicotyledons (*i.e.*, cellulose, lignin, and *4-O-methyl glucuronoxylan*) were discovered within QTNs related to the chemical composition of the biomass. These polymers are the most abundant constituent material of the plant cell walls, thus constituting the major components of plant biomass. They interact with themselves and with each other via covalent and noncovalent bonds to form a macromolecular network that determines the biological and physical properties of the secondary wall. Here, we detected a *Cellulose synthase* (*CESA*)*,* a *Glucuronoxylan 4-O-methyltransferase,* and three different peroxidases associated with SCSa, ADF and NDF, respectively. In Arabidopsis, *CESA1*, *CESA3* and *CESA6* (or *CESA6-like*) are required for primary wall cellulose synthesis. Chu et al. [62] observed that the knockout of *AtCESA2* caused severe defects in cell wall formation that led to abnormal plant growth and development. By contrast, the transgenic lines overexpressing *CESA2* showed enhanced growth performance with increased biomass production. Similarly, *PmCESA2* in poplar led to an altered cell wall polysaccharide composition, which resulted in the thickening of the secondary cell wall and xylem width [63]. Consequently, the cellulose and lignin content were increased. Consistent with these studies, *CESA* could be used as a potential candidate gene to enhance cellulose synthesis and biomass accumulation in wheat. Coincident with the role of *CESA*, genes encoding secondary cell wall xylan and its modifications (i.e., *GXMT*) are also important for biomass production [64, 65]. Since genetic approaches have provided limited insight into the mechanisms of 4-O-methyl glucuronoxylan synthesis, our candidate gene annotated as *Glucuronoxylan 4-O-methyltransferase* may represent a new target to selectively manipulate polysaccharide O-methylation, providing new opportunities to modulate biopolymer interactions in the wheat cell wall. It is noteworthy that the presence of lignin in cell walls is also important since it imparts recalcitrance in the deconstruction of the wall materials for pulping and biofuel production [66, 67]. To reduce cell wall recalcitrance, a great deal of interest has been invested in engineer lignin and its composition (Van Acker et al. 2014, [20, 21, 68]. In model plants, down-regulation or silencing of genes (*PRX2*, *PRX3*, *PRX22*, *PRX60*, *PRX71*, and *PRX72*) encoding peroxidases resulted in reduced lignin accumulation and altered lignin composition [69, 70]. Consistent with these studies, in our work, we found three different peroxidases (*PRX1*, *PRX22,* and *PRX36*) that might be important for lignin production and/or its degradation.

Despite the well-known genes reported above, other candidate genes with a role in cell architecture, plant growth regulators, photosynthetic pathways, and microRNA biogenies were also found. For example, an *Anaphase promoting complex* (*APC*/*C*) was significantly associated with CEL trait (*Q.Cel-2A*). It has been shown that when the *Arabidopsis APC3a*/*CDC27a* gene is overexpressed in tobacco, it accelerated plant growth, leading to plants with increased biomass [71]. Similar results were also obtained when tobacco plants overexpressing the *APC10* gene from *Arabidopsis* increased biomass and reduced life cycle length [72]. Another interesting candidate is *HYPONASTIC LEAVES 1* (*HYL1*). This gene encodes a nuclear double-stranded RNA-binding protein which is involved in microRNA (miRNA) biogenesis, and in the regulation of miR156 [73, 74]. The overexpression of miR156 in *Arabidopsis* caused increased total leaf numbers, and biomass [75]. Similarly, alfalfa plants overexpressing miR156 had reduced internode length and stem thickness and elevated biomass production [76]. In red clover, overexpression of miR156 increased the number of shoots, delayed flowering, and accelerated biomass accumulation [77].

In addition, we also found a *Transaldolase* (*TAL*) within the region flanking the QTN *Q.Spl-3B*. Chen et al. [78] in *Pichia stipites* identified a *TAL* gene as a rate-limiting enzyme for xylose-to-ethanol bioconversion. Indeed, despite the increase in the understanding of the molecular mechanisms involved in biomass production and composition, it is also important to consider the conversion of biomass products to biofuel. Using overexpressed lines Chen et al. [78] reported an increase in ethanol production by 36% and 100%, suggesting that improving the *Transaldolase* activity in *P. stipitis* can significantly increase the rate and yield of xylose conversion to ethanol. Thus, the identified superior alleles with significant effect in the present study (*i.e.*, those for ADF, NDF, CEL, and SCSa) may have critical role for improving biomass composition in wheat varieties with positive effects on bioethanol production.

## Conclusions

Our study will provide new insights to the genetic basis of biomass composition traits in tetraploid wheat. The application of six ML-GWAS models on a panel of diverse wheat genotypes provided an efficient approach to dissect complex traits with low heritability such as wheat straw composition. A total of 72 reliable QTNs were detected by two or more than two models. Among the major QTNs identified in this study, 16 QTNs showed a significant effect on the corresponding phenotypes. Further, putative candidate genes were identified from the associated genomic region. In addition, a marker associated with SCSb has been validated through molecular screening (HRM and rhAmp), providing a reliable marker for MAS applications. The discovery of genes/genomic regions associated with biomass production and straw quality parameters is expected to accelerate the development of high-producing wheat varieties useful for biofuel production. The information generated in this study would be also useful as a basis for further functional investigation especially in the genomic region close the validated marker and define a new wheat ideotype.

## Methods

### Pant materials and field experiments

The tetraploid wheat (*Triticum turgidum* L., 2n = 4x = 28; AABB genome) collection used in this study was comprised of 185 accessions available in the germplasm bank at CREA Research Centre for Cereal and Industrial Crops in Foggia. The panel, including wild, domesticated and cultivated accessions of seven subspecies (*dicoccoides, dicoccum, carthlicum*, *polonicum, turanicum*, *turgidum*, and *durum*), was chosen to represent a wide phenotypic variability for the main morphological traits that were evaluated in this study.

The wheat collection was grown in southern Italy at the experimental farm of CREA Research Centre for Cereal and Industrial Crops at Foggia (41°27′36″ N, 15°30′05″ E) for three growing-seasons (2009, 2010, 2012) on a clay-loam soil (Typic Chromoxerert), with the following main chemical characteristics: organic matter (Walkley–Black method) 2.5 and 2.6%; available phosphorus (Olsen method) 62.0 and 68.0 mg kg^−1^; exchangeable potassium (ammonium acetate method) 422 and 450 mg kg^−1^; total nitrogen (Dumas method) 1.3 and 1.1%. The genotypes were sown on recommended dates and arranged in randomized complete blocks with 2 replications. Plots comprised eight rows of 7.5 m in length with a distance between rows of 0.17 m. The sowing density was always 350 seeds m^2^. The field experiments were supplied with 45 kg/ha N and 115 kg/ha P_2_O_5_ as pre-sowing and 85 kg/ha N as top dressing each year. Weeds, pests, and fungal diseases were chemically controlled.

### Morphological traits

Plant height (PH) (in centimeters) was measured during the milk-waxy maturation when the maximum height level was achieved, from ground to the tip of the ear (excluding awns) on five main culms per plot. To evaluate stem solidness, more than 5 stems were randomly selected at post-anthesis and were cross-sectionally cut at the center of each internode in the basal (SCSb), median (SCSm), and apical (SCSa) part of each stem. The level of stem solidity was rated as 1–5 (1 for hollow and 5 for solid) using the UPOV scoring system [79]. At physiological maturity, above-ground dry matter was determined by cutting plants at the soil surface from a 1 m^2^ area (6 rows × 0.95 m). The plants collected were oven-dried at 70 °C for 48 h and weighed for total dry matter. Then, the spikes were cut, measured in length (SPL, cm), and threshed, and the grain was weighed (GW). Straw dry weight (Biomass) was calculated as the difference between above-ground biomass and grain weight. Harvest index (HI) was calculated as was calculated as the ratio of grain weight to total biomass. Trait acronyms are reported in Supplementary Table 3.

### Cell-wall chemical analysis

Cell-wall carbohydrates were quantified by determination of acid detergent fiber (ADF), acid detergent lignin (ADL), and neutral detergent fiber (NDF) according to the methods of Van Soest et al. [80] using an ANKOM 220 Fibre Analyzer (ANKOM Technology Corporation, NY, USA). Hemicellulose was calculated as NDF – ADF and cellulose as ADF – ADL [81]. Trait acronyms are reported Supplementary Table 3.

### DNA material and Plant genotyping

Genetic variation data, generated using the Illumina  wheat 90 K iSelect Assay developed by TraitGenetics [82], were extracted from a bigger population deposited at Mendeley Data website (https://data.mendeley.com) with the following DOI number: 10.17632/rt2gmzbvmz.1. The whole dataset can be downloaded using the link (https://data.mendeley.com/datasets/rt2gmzbvmz/1). The raw dataset related to the 185 genotypes under study was processed with plink [83] using a call rate value lower than 95% and a minimum allele frequency (MAF) lower than 5%. After filtering, a total number of 20,755 SNPs was used for the downstream analysis. The resulting VCF file related to only 185 individuals under study is available at the Figshare data repository (https://figshare.com) under the following DOI number: 10.6084/m9.figshare.18586076. Data can be downloaded using the following link: https://doi.org/10.6084/m9.figshare.18586076.v1.

Principal component analysis (PCA) was calculated usng the resulting SNPs & Variation suite (SVS) v.8.4.0 (Golden Helix inc) and drawn in R [84].

### Multi-locus genome-wide association analysis

Association analysis was performed using multi-locus random-SNP-effect MLM, (mrMLM) [24], fast mrMLM (FASTmrMLM) [85], iterative modified-sure independence screening expectation–maximization-Bayesian least absolute shrinkage and selection operator (ISIS EM-BLASSO) [23], integration of Kruskal–Wallis test with empirical Bayes (pKWmEB) [86], fast multi-locus random-SNP-effect efficient mixed model analysis (FASTmrEMMA) [87], and polygenic-background-control-based least angle regression plus empirical Bayes (pLARmEB) [27]. All ML-GWA models were tested by using mrMLM v4.0 [28], downloaded from http://cran.r-project.org/web/packages/mrMLM/index.html. Kinship matrix was calculated by the specific option implemented in the mrMLM v4.0 package [28] and used in all methods as covariate. Default values were used for all parameters. In particular, the REML option for the Likelihood Function was used for FASTmrEMMA model, whereas the bootstrapping was chosen for pLARmEB model. The association analysis was conducted using two approaches: i) Kinship matrix, ii) K + PCA as Q matrix. As proposed by Zhang et al. [88] for multi-locus GWA analysis, we used a LOD = 3.0 (or *P* = 0.0002) as a cut-off to balance the high power and low false positive rate for QTN detection. In addition, SNP markers detected by two or more different models were designated as reliable QTNs, as suggested by Chaurasia et al. [25]. QTNs with *r*^2^ values > 10% were declared as major, as also showed by Chaurasia et al. [25].

### Principal component (PCA), analysis of variance (ANOVA), Broad-sense heritability (H^2^) and Pearson correlation

A two-way analysis of variance (ANOVA) was implemented to investigate the genotype and year effects, their interaction (genotype x year) and residuals. Broad-sense heritability (H^2^) was estimated as follows:

1 $$\mathrm H^2=\sigma \mathrm{g}/\;\left[\sigma \mathrm{g}+\left(\sigma\mathrm{gy}/\mathrm y\right)+\left(\sigma e/\tau\mathrm{y}\right)\right]$$

where σg is the genotypic variance, σgy the variance explained by the interaction between genotypes and year, σe the variance of residuals, τ the number of the replicates and y the number of the year. Best linear unbiased prediction (BLUP) of phenotypic traits collected over years were calculated using the following mixed linear model:

$${\mathrm y}_{\mathrm i}\mathrm j\:=\:\mathrm\mu+{\mathrm g}_{\mathrm i}\:+{\mathrm t}_{\mathrm j}+\;\left[\mathrm{gt}\right]\;_{\mathrm i}\mathrm j+\mathrm e$$

where y_ij are the observed traits, μ is the overall mean, g_i is the effect of the ith line assumed as random effect, t_j is the effect of the jth trial (year) modelled as random effect, [gt]_ij are the genotype-trial interaction, and e corresponds to the residual effect considering as random and assuming to have a normal distribution r ~ N(0,[Iσ]_r^2). The model was implemented using the function lmer in the R package lme4 [89]. The normal distribution of BLUP data was verified using the Shapiro test. In addition, principal component analysis (PCA) was performed with BLUP values. ANOVA, BLUPs, PCA, and correlation analyses (Pearson’s correlation with significance level α = 0.05) were carried out using FactoMinerR [90], Lme4 R [89], factoextra [91], and corrplot [92] packages.

### Candidate genes

Putative candidate genes were searched in flanking regions of the significant QTNs. The linkage disequilibrium (LD) decay value was calculated using the LD Ajacent Pairs Analysis function (SVS) and then used to define the confidence interval.

Then, gene annotation was retrieved based on the Svevo durum wheat high-confidence gene models (https://www.interomics.eu/durum-wheat-genome). Putative candidates were then used as baits for a BLASTn search against the NCBI database to assign gene names based on direct orthologs of *Oryza sativa*.

### SNP marker assay validation

Firstly, two different molecular methods (High-Resolution Melting analysis (HRM) and rhAmp allelic discrimination assay) have been used to validate the SNP marker associated with basal stem solidness using a panel of 34 accessions with a contrasting behavior for SCSb and for allelic profiles. As far as HRM is concerning, primer3 software version 4.0.0 (Whitehead Institute for Biomedical Research, Cambridge, MA; http://primer3.ut.ee) was adopted to design primers. The HRM analyses were performed in 384 well plates on the QuantStudio 12 K Flex (Life Technologies, USA), following the procedure described by [93], whereas the rhAmp allelic discrimination assay was carried out following the procedure described by Broccanello et al. [94] and Ravi et al. [93]. Sequences of rhAmp assays are available upon request.   

## Supplementary Information

**Additional file 1: Supplementary  Fig. 1. **Frequency distribution of BLUPsfor 15 traits among cultivars. X-axisshow BLUP values while in the y-axisthe frequency is reported. 

**Additional file 2:** **Supplementary Fig. 2.** Boxplot showing phenotypic distribution among different wheat subspecies.Statistical significant differences are shown with different letters. 

**Additional file 3: Supplementary  Fig. 3. **Genotypic variability of the 185wheat genotypes. Loading plot of the first (PC1) and second (PC2) principalcomponents showing the variation among individuals. Based on Triticum ssp.,genotypes are represented by different colored symbols indicated in the legend.

**Additional file 4: Supplementary  Fig. 4. **Intra-chromosomal LD decaydistance (kb) evaluated considering (A) the whole genome, (B) A genome, (C) Bgenome. Dashed lines indicate the r2 threshold. The intersectionpoint between the decay LD curve and the LD threshold was shown. 

**Additional file 5: Supplementary Fig. 5. **Numbers of significant QTNs detected for 15 traits using six multi-locus GWASmethods 

**Additional file 6: Supplementary Fig. 6. **Singular QQ plots for associatedtraits. On X axis the expected –log10pvalues, whereas observed –log10pvalues are reported on Y axis. 

**Additional file 7: Supplementary Table 1.** BLUPs values for the traits under study.

**Additional file 8:** **Supplementary Table 2.** Molecular validation of RFL_Contig3228_2154)associated with SCSb with HRM and rhAMP assays.

**Additional file 9:** **Supplementary Table  3.** List of acronyms and their abbreviation used in this study.

**Additional file 10: Supplementary Table 4. **QTNs comparison with QTL identified in the literature.

## Data Availability

The VCF dataset generated and analyzed during the current study is available in the Figshare repository (https://figshare.com/) under the following DOI number: 10.6084/m9.figshare.18586076. Data can be downloaded using the following link: https://doi.org/10.6084/m9.figshare.18586076.v1. The whole dataset is available at Mendeley Data website (https://data.mendeley.com) with the following DOI number:; and it can be downloaded using the link: https://data.mendeley.com/datasets/rt2gmzbvmz/1.

## Abbreviations

ML-GWAS: Multi-locus genome-wide association study
QTNs: Quantitative trait nucleotides
HRM: High resolution melting
RhAMP: RNase H2-dependent PCR
MAF: Minimum allele frequency
ADF: Acid detergent fiber
ADL: Acid detergent lignin
NDF: Neutral detergent fiber
SCSa: Straw cross section apical
SCSb: Straw cross section basal
SCSm: Straw cross section medium
GW: Grain weight
SPL: Spike length
TTN: Culm numbers
FTN: Spikes number
CEL: Cellulose
Biomass: Total Biomass
HEM: Hemicellulose
PH: Plant Height
HI: Harvest index
