# Supplementary material for: Unlocking the molecular basis of wheat straw composition and morphological traits through multi-locus GWAS
Source: BMC Plant Biol. 2022 Nov 8;22:519. doi: 10.1186/s12870-022-03900-6 (PMC9641881; doi:10.1186/s12870-022-03900-6)
Supplement: Supplementary file 1 — Additional file 1: Supplementary Fig. 1. Frequency distribution of BLUPsfor 15 traits among cultivars. X-axisshow BLUP values while in the y-axisthe frequency is reported. [file 12870_2022_3900_MOESM1_ESM.pptx]

## Slide 1
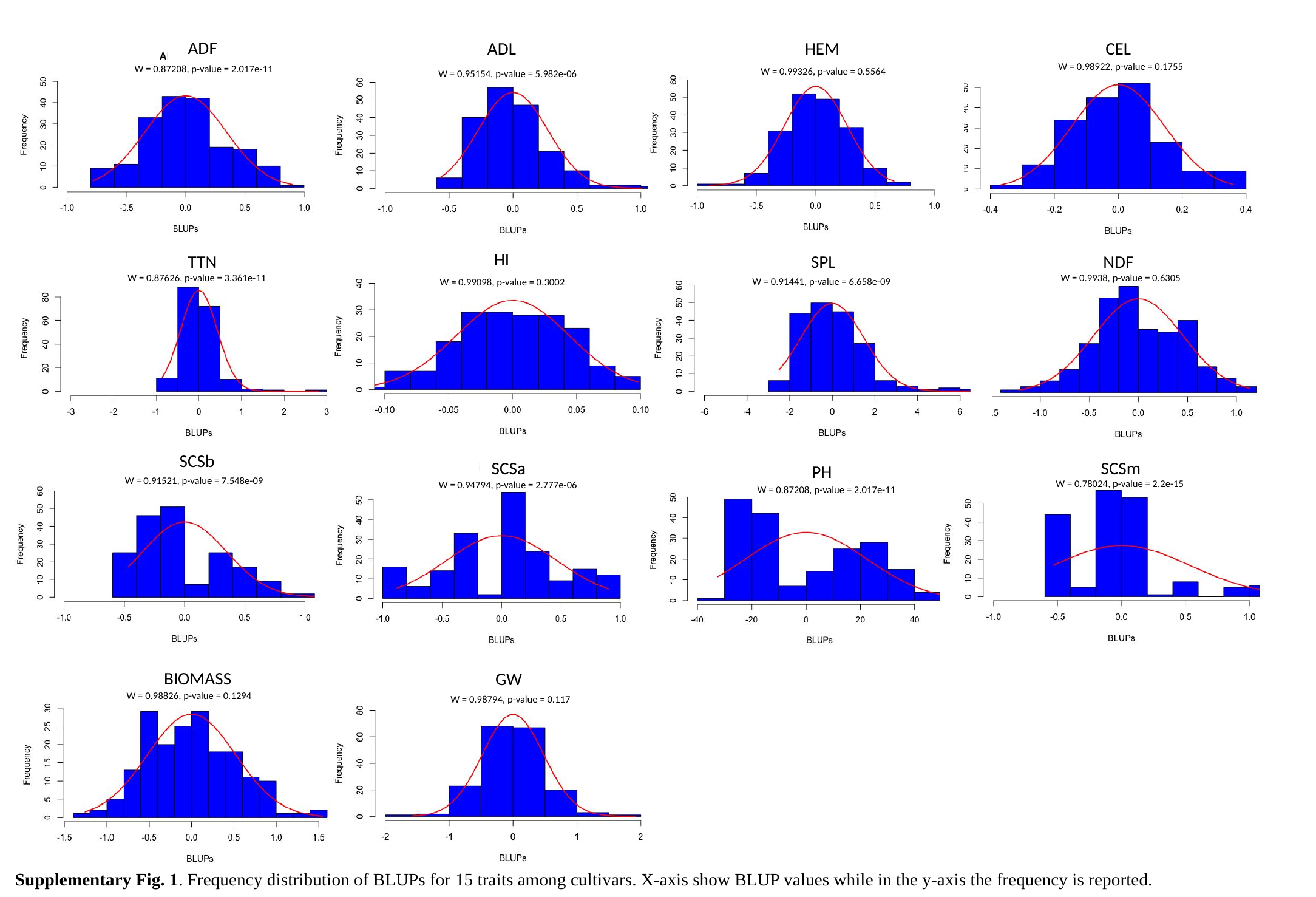

ADF
ADL
HEM
CEL
ADF
ADF
ADF
ADF
W = 0.98922, p-value = 0.1755
W = 0.87208, p-value = 2.017e-11
W = 0.99326, p-value = 0.5564
W = 0.95154, p-value = 5.982e-06
HI
TTN
NDF
SPL
ADF
ADF
ADF
ADF
W = 0.87626, p-value = 3.361e-11
W = 0.9938, p-value = 0.6305
W = 0.91441, p-value = 6.658e-09
W = 0.99098, p-value = 0.3002
SCSb
ADF
ADF
SCSa
SCSm
TTN
SCSm
ADF
PH
TTN
W = 0.91521, p-value = 7.548e-09
W = 0.78024, p-value = 2.2e-15
W = 0.94794, p-value = 2.777e-06
W = 0.87208, p-value = 2.017e-11
BIOMASS
GW
TTN
TTN
W = 0.98826, p-value = 0.1294
W = 0.98794, p-value = 0.117
Supplementary Fig. 1. Frequency distribution of BLUPs for 15 traits among cultivars. X-axis show BLUP values while in the y-axis the frequency is reported.
