# Supplementary material for: Unlocking the molecular basis of wheat straw composition and morphological traits through multi-locus GWAS
Source: BMC Plant Biol. 2022 Nov 8;22:519. doi: 10.1186/s12870-022-03900-6 (PMC9641881; doi:10.1186/s12870-022-03900-6)

## Slide 1
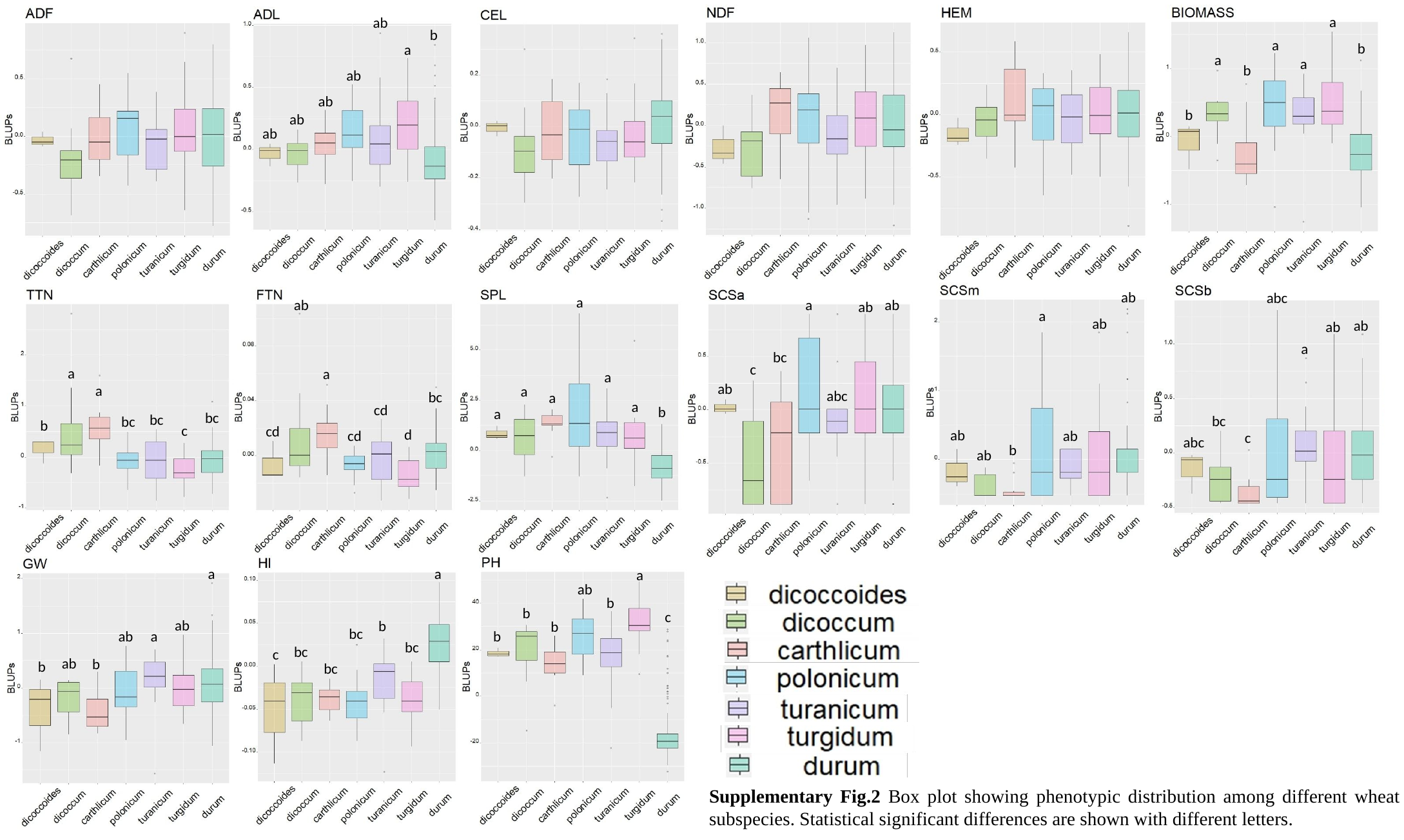

a
ab
b
a
b
a
a
a
b
ab
ab
b
ab
ab
ab
abc
a
ab
a
ab
ab
a
ab
ab
ab
a
bc
c
a
a
a
ab
a
a
abc
bc
a
a
cd
b
a
bc
bc
bc
bc
b
c
cd
d
ab
ab
cd
c
abc
b
ab
a
a
a
ab
b
b
c
ab
b
b
bc
a
b
ab
bc
bc
c
ab
b
b
bc
Supplementary Fig.2 Box plot showing phenotypic distribution among different wheat subspecies. Statistical significant differences are shown with different letters.

## Slide 2
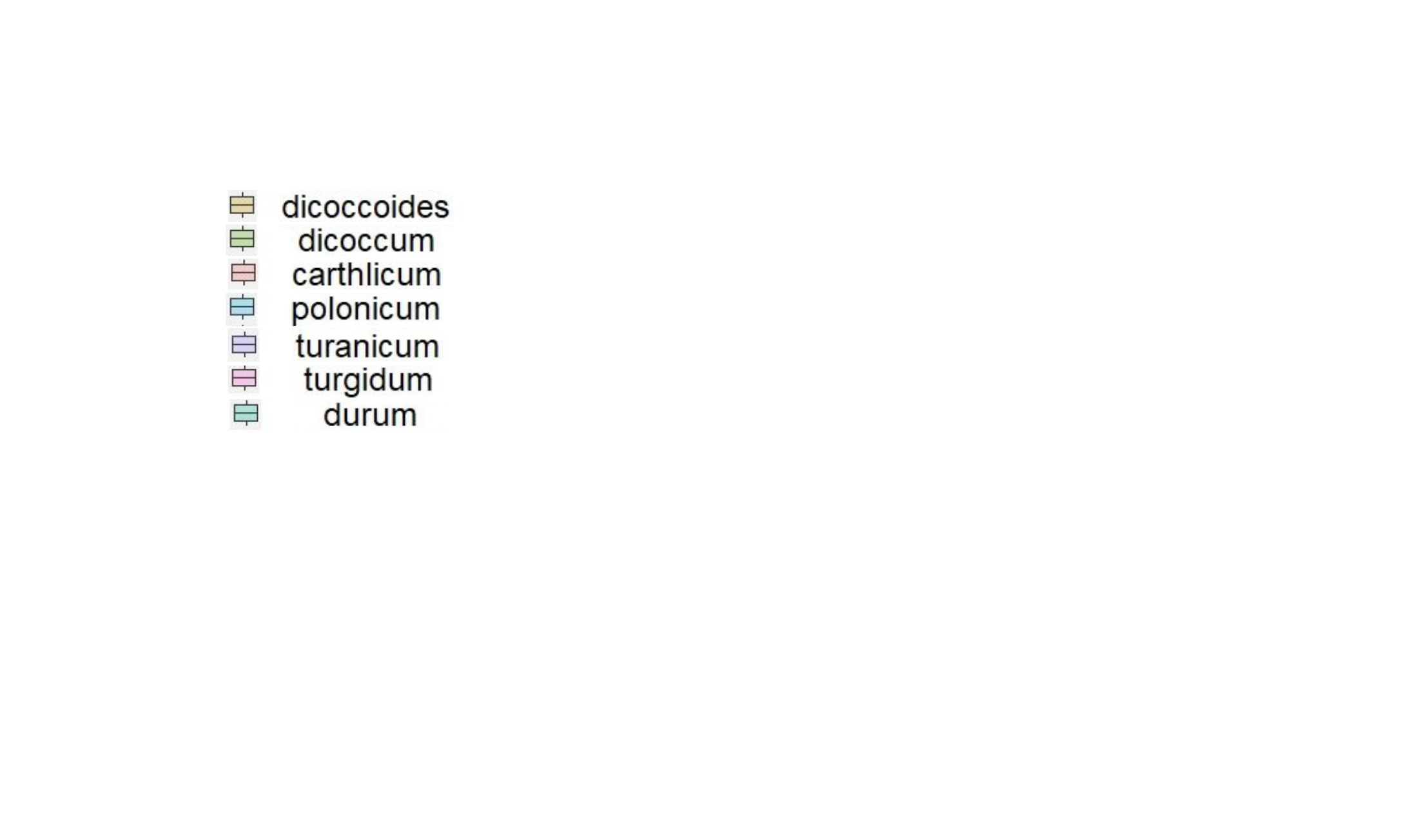

Supplement: Supplementary file 2 — Additional file 2: Supplementary Fig. 2. Boxplot showing phenotypic distribution among different wheat subspecies.Statistical significant differences are shown with different letters. [file 12870_2022_3900_MOESM2_ESM.pptx]
