# Supplementary material for: Unlocking the molecular basis of wheat straw composition and morphological traits through multi-locus GWAS
Source: BMC Plant Biol. 2022 Nov 8;22:519. doi: 10.1186/s12870-022-03900-6 (PMC9641881; doi:10.1186/s12870-022-03900-6)
Supplement: Supplementary file 3 — Additional file 3: Supplementary Fig. 3. Genotypic variability of the 185wheat genotypes. Loading plot of the first (PC1) and second (PC2) principalcomponents showing the variation among individuals. Based on Triticum ssp.,genotypes are represented by different colored symbols indicated in the legend. [file 12870_2022_3900_MOESM3_ESM.pptx]

## Slide 1
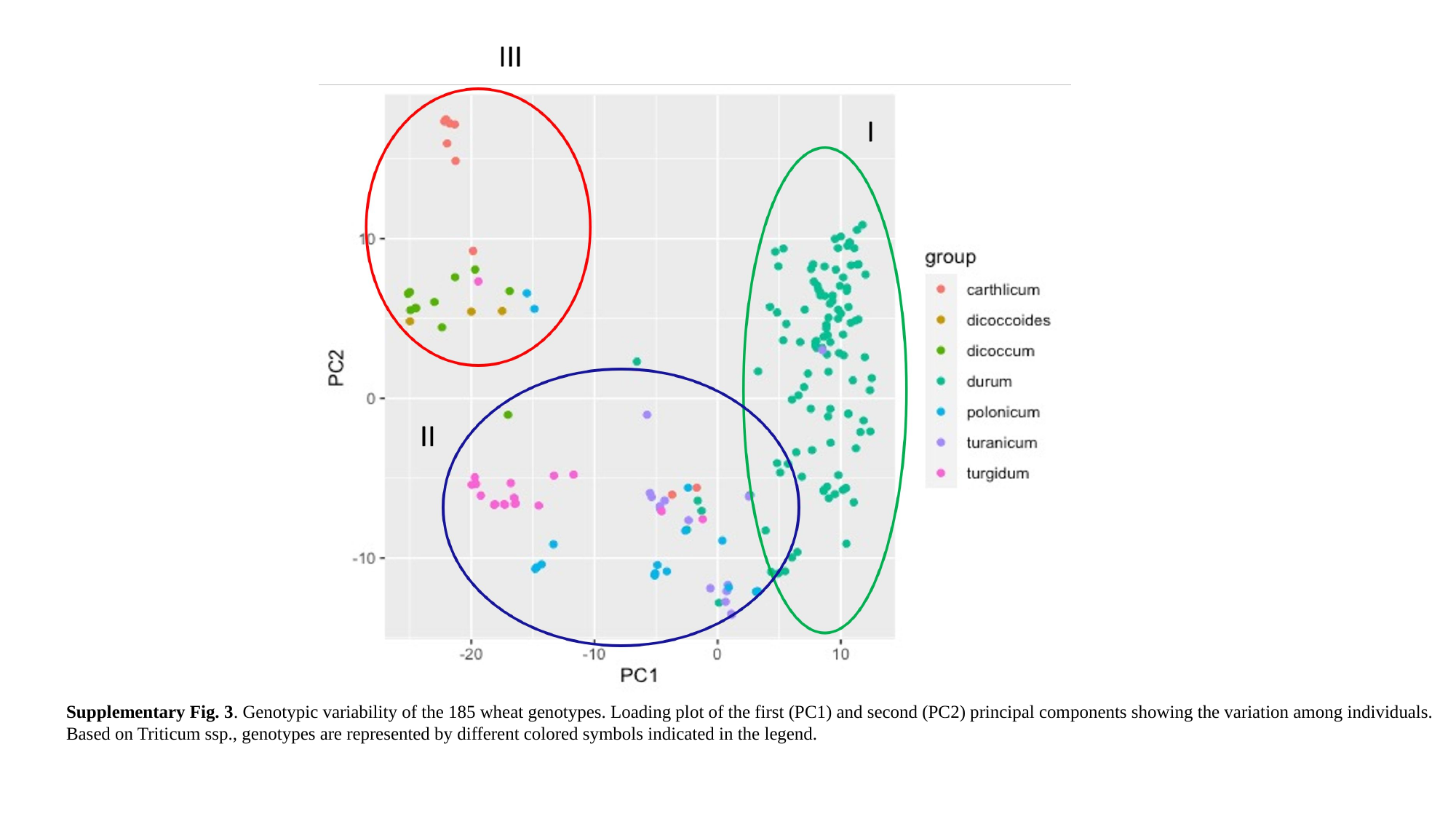

Supplementary Fig. 3. Genotypic variability of the 185 wheat genotypes. Loading plot of the first (PC1) and second (PC2) principal components showing the variation among individuals. Based on Triticum ssp., genotypes are represented by different colored symbols indicated in the legend.
