# Supplementary material for: Unlocking the molecular basis of wheat straw composition and morphological traits through multi-locus GWAS
Source: BMC Plant Biol. 2022 Nov 8;22:519. doi: 10.1186/s12870-022-03900-6 (PMC9641881; doi:10.1186/s12870-022-03900-6)
Supplement: Supplementary file 4 — Additional file 4: Supplementary Fig. 4. Intra-chromosomal LD decaydistance (kb) evaluated considering (A) the whole genome, (B) A genome, (C) Bgenome. Dashed lines indicate the r2 threshold. The intersectionpoint between the decay LD curve and the LD threshold was shown. [file 12870_2022_3900_MOESM4_ESM.pptx]

## Slide 1
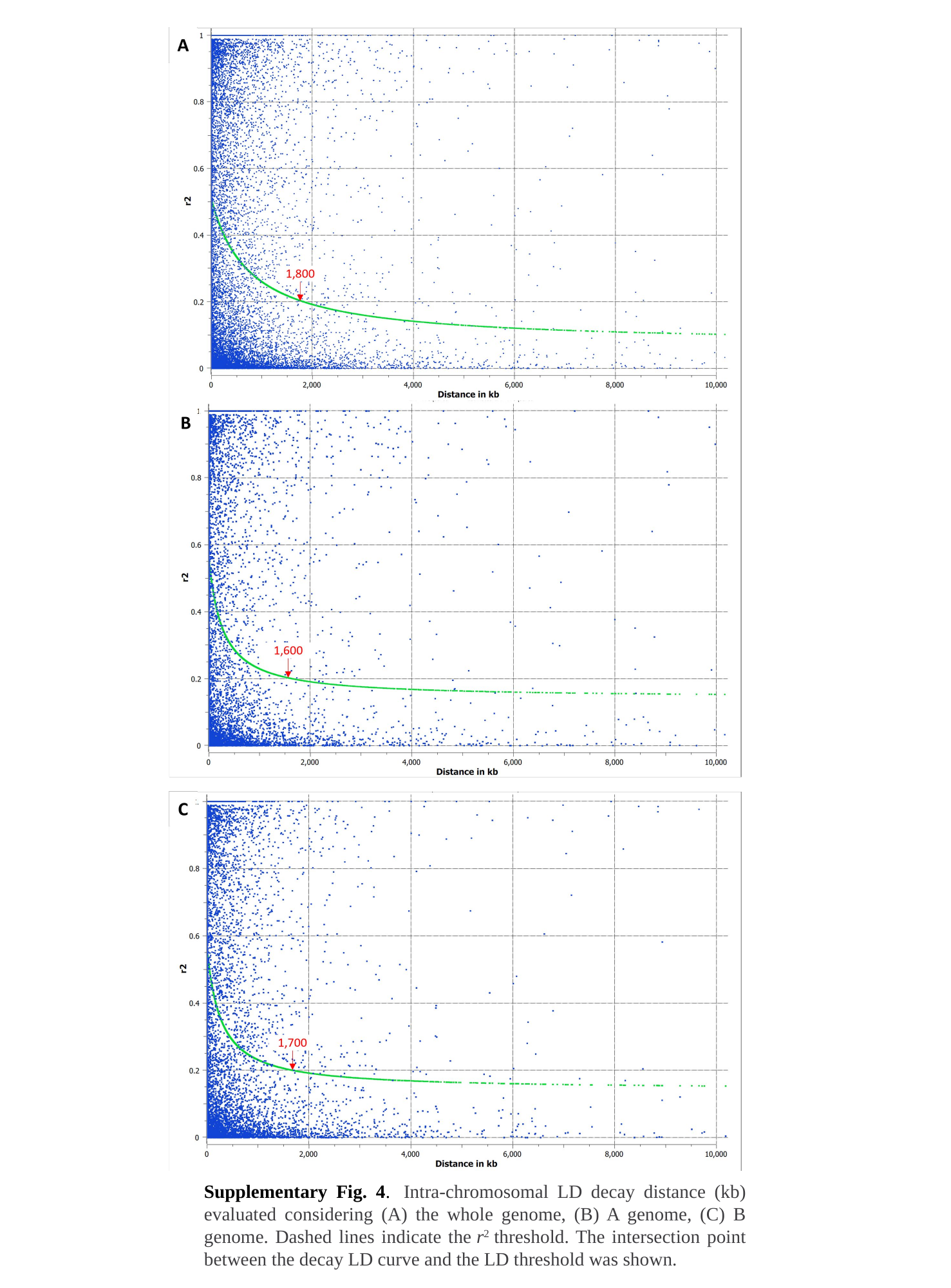

Supplementary Fig. 4.  Intra-chromosomal LD decay distance (kb) evaluated considering (A) the whole genome, (B) A genome, (C) B genome. Dashed lines indicate the r2 threshold. The intersection point between the decay LD curve and the LD threshold was shown.
