# Supplementary material for: Unlocking the molecular basis of wheat straw composition and morphological traits through multi-locus GWAS
Source: BMC Plant Biol. 2022 Nov 8;22:519. doi: 10.1186/s12870-022-03900-6 (PMC9641881; doi:10.1186/s12870-022-03900-6)
Supplement: Supplementary file 5 — Additional file 5: Supplementary Fig. 5. Numbers of significant QTNs detected for 15 traits using six multi-locus GWASmethods [file 12870_2022_3900_MOESM5_ESM.pptx]

## Slide 1
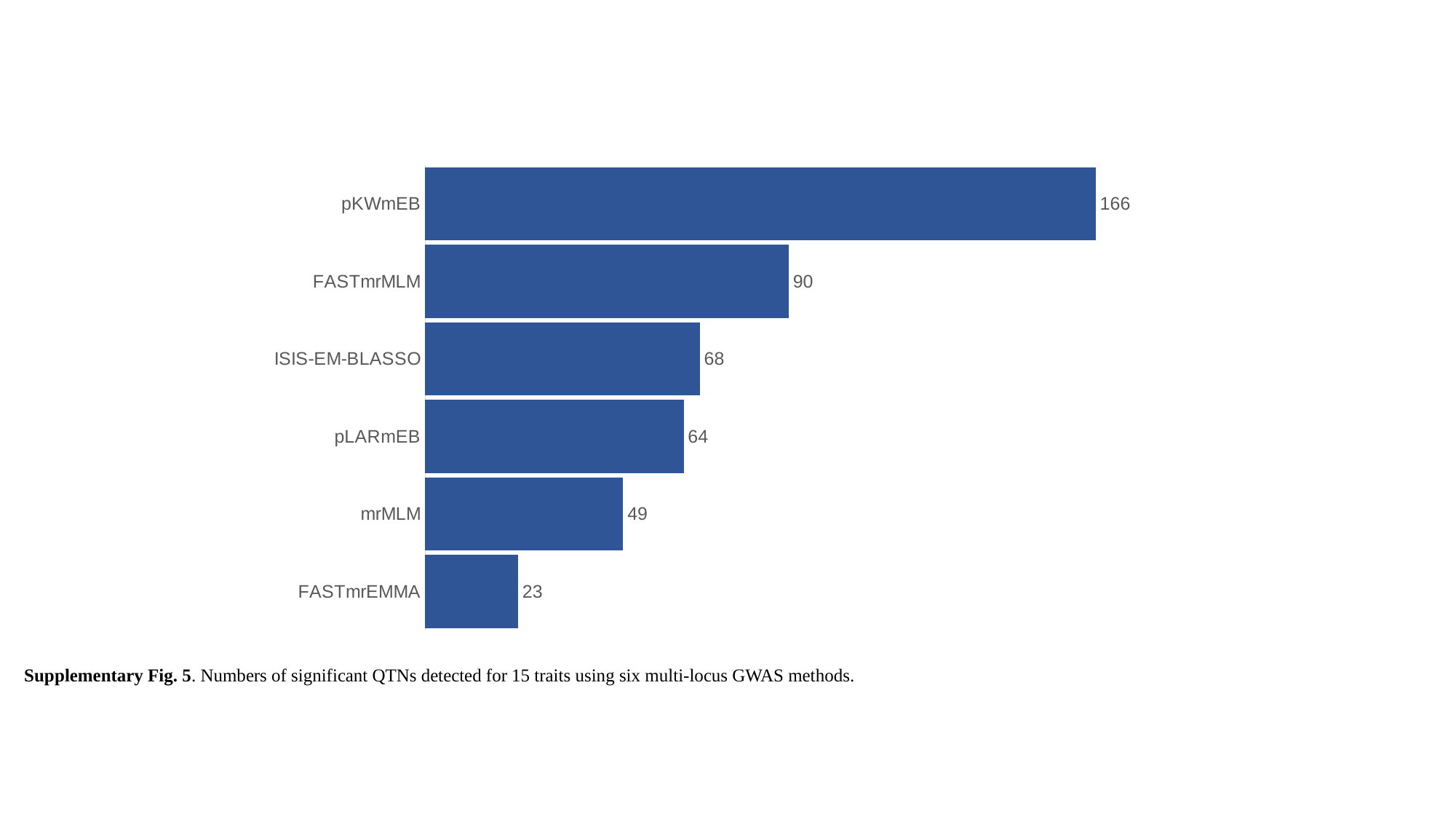

### Chart
| Category | |
|---|---|
| FASTmrEMMA | 23.0 |
| mrMLM | 49.0 |
| pLARmEB | 64.0 |
| ISIS-EM-BLASSO | 68.0 |
| FASTmrMLM | 90.0 |
| pKWmEB | 166.0 |Supplementary Fig. 5. Numbers of significant QTNs detected for 15 traits using six multi-locus GWAS methods.
