# Supplementary material for: Unlocking the molecular basis of wheat straw composition and morphological traits through multi-locus GWAS
Source: BMC Plant Biol. 2022 Nov 8;22:519. doi: 10.1186/s12870-022-03900-6 (PMC9641881; doi:10.1186/s12870-022-03900-6)
Supplement: Supplementary file 6 — Additional file 6: Supplementary Fig. 6. Singular QQ plots for associatedtraits. On X axis the expected –log10pvalues, whereas observed –log10pvalues are reported on Y axis. [file 12870_2022_3900_MOESM6_ESM.pptx]

## Slide 1
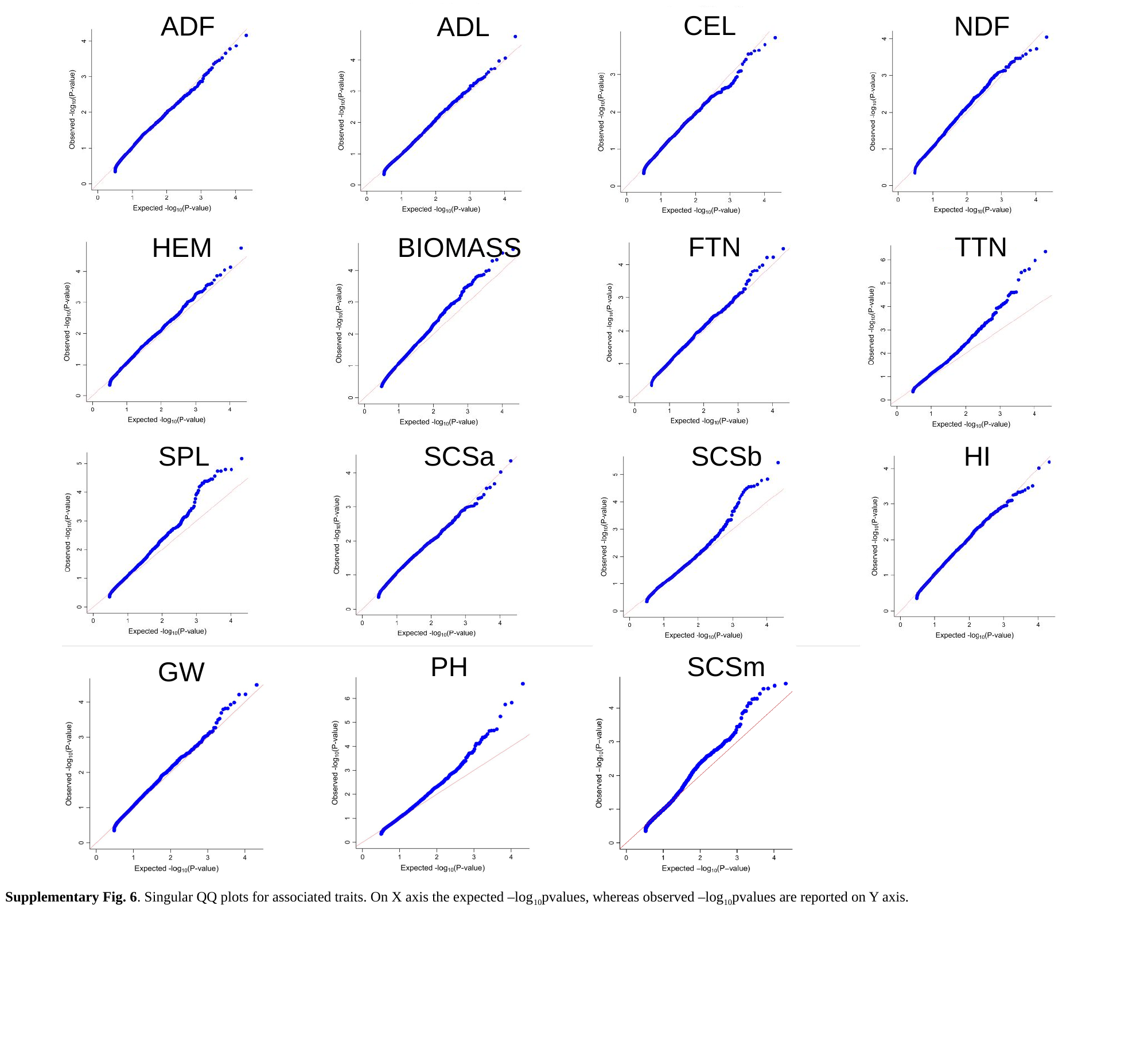

HI
SPL
TTN
SCSa
CEL
ADF
NDF
ADL
TTN
TTN
TTN
TTN
BIOMASS
GW
HEM
ADF
TTN
TTN
FTN
TTN
TTN
HEM
BIOMASS
TTN
SPL
SCSa
SCSb
HI
TTN
CEL
NDF
ADL
TTN
TTN
TTN
PH
SCSm
GW
Supplementary Fig. 6. Singular QQ plots for associated traits. On X axis the expected –log10pvalues, whereas observed –log10pvalues are reported on Y axis.
