# Supplementary material for: Unlocking the molecular basis of wheat straw composition and morphological traits through multi-locus GWAS
Source: BMC Plant Biol. 2022 Nov 8;22:519. doi: 10.1186/s12870-022-03900-6 (PMC9641881; doi:10.1186/s12870-022-03900-6)
Supplement: Supplementary file 8 — Additional file 8: Supplementary Table 2. Molecular validation of RFL_Contig3228_2154)associated with SCSb with HRM and rhAMP assays. [file 12870_2022_3900_MOESM8_ESM.docx]

| **Taxa** | **HRM** | **rhAMP** |
| --- | --- | --- |
| PI-349051^a^ | variant2 | Homozygous Allele 2/Allele 2 |
| PI-134946^a^ | variant2 | Homozygous Allele 2/Allele 2 |
| PI-576854^a^ | variant2 | Homozygous Allele 2/Allele 2 |
| Quadruro^a^ | variant2 | Homozygous Allele 2/Allele 2 |
| Aziziah^a^ | variant2 | Homozygous Allele 2/Allele 2 |
| K26 | variant2 | Homozygous Allele 2/Allele 2 |
| PI-499972^b^ | variant1 | Homozygous Allele 1/Allele 1 |
| Farvento^b^ | NA | NA |
| MG-4387^b^ | variant1 | Homozygous Allele 1/Allele 1 |
| MG5344/1^b^ | variant1 | Homozygous Allele 1/Allele 1 |
| MG5293/1^b^ | variant1 | Homozygous Allele 1/Allele 1 |
| Capeiti-8^b^ | variant1 | Homozygous Allele 1/Allele 1 |
| MG-5444^b^ | NA | NA |
| PI-341800^b^ | NA | NA |
| PI-306549^b^ | variant1 | Homozygous Allele 1/Allele 1 |
| PI-341391^b^ | variant1 | Homozygous Allele 1/Allele 1 |
| PI-352489^b^ | variant1 | Homozygous Allele 1/Allele 1 |
| PI-576854^b^ | NA | NA |
| Italo^b^ | variant1 | Homozygous Allele 1/Allele 1 |
| AncoMarzo | NA | NA |
| PI-352488^b^ | variant1 | Homozygous Allele 1/Allele 1 |
| PI-566593^b^ | variant1 | Homozygous Allele 1/Allele 1 |
| Platani^b^ | variant1 | Homozygous Allele 1/Allele 1 |
| Iride^b^ | variant1 | Homozygous Allele 1/Allele 1 |
| Ofanto^b^ | variant1 | Homozygous Allele 1/Allele 1 |
| Orobel^b^ | variant1 | Homozygous Allele 1/Allele 1 |
| PI-355459^b^ | variant1 | Homozygous Allele 1/Allele 1 |
| Sansone^b^ | variant1 | Homozygous Allele 1/Allele 1 |
| Saragolla^b^ | variant1 | Homozygous Allele 1/Allele 1 |
| Svevo^b^ | variant1 | Homozygous Allele 1/Allele 1 |
| Trinakria^b^ | variant1 | Homozygous Allele 1/Allele 1 |
| MG5473^b^ | NA | NA |
| Exeldur^b^ | variant1 | Homozygous Allele 1/Allele 1 |
| Fiore^b^ | variant1 | Homozygous Allele 1/Allele 1 |

**Supplementary Table 2.** Molecular validation of RFL_Contig3228_2154) associated with SCSb with HRM and rhAMP assays.

a= high Scsb; b= low Scsb
