# Supplementary material for: Unlocking the molecular basis of wheat straw composition and morphological traits through multi-locus GWAS
Source: BMC Plant Biol. 2022 Nov 8;22:519. doi: 10.1186/s12870-022-03900-6 (PMC9641881; doi:10.1186/s12870-022-03900-6)
Supplement: Supplementary file 9 — Additional file 9: Supplementary Table 3. List of acronyms and their abbreviation used in this study. [file 12870_2022_3900_MOESM9_ESM.docx]

| **Trait** | **Description** |
| --- | --- |
| ADF | acid detergent fiber |
| ADL | acid detergent lignin |
| NDF | neutral detergent fiber |
| SCSa | straw cross section apical |
| SCSb | straw cross section basal |
| SCSm | straw cross section medium |
| GW | grain weight |
| SPL | spike lenght |
| TTN | culm numbers |
| FTN | spikes number |
| CEL | Cellulose |
| Biomass | Total Biomass |
| HEH | Hemicellulose |
| PH | Plant Height |
| HI | Harvest index |

**Supplementary Table 3**. List of acronyms and their abbreviation used in this study.
